# Identification of a molecular signature of prognostic subtypes in diffuse-type gastric cancer

**DOI:** 10.1007/s10120-019-01029-4

**Published:** 2019-11-26

**Authors:** Seon-Kyu Kim, Hee-Jin Kim, Jong-Lyul Park, Haejeong Heo, Seon-Young Kim, Sang-Il Lee, Kyu-Sang Song, Woo-Ho Kim, Yong Sung Kim

**Affiliations:** 1 Personalized Genomic Medicine Research Center, Korea Research Institute of Bioscience and Biotechnology, Daejeon, 34141 Korea; 2 Genome Editing Research Center, Korea Research Institute of Bioscience and Biotechnology, 125 Gwahak-ro, Yuseong-gu, Daejeon, 34141 Korea; 3 Department of Bioscience, University of Science and Technology, Daejeon, 34113 Korea; 4 Department of General Surgery, College of Medicine, Chungnam National University, Daejeon, 35015 Korea; 5 Department of Pathology, College of Medicine, Chungnam National University, Daejeon, 35015 Korea; 6 Department of Pathology, Faculty of Medicine, Seoul National University, Seoul, 03080 Korea

**Keywords:** Gastric cancer, Diffuse-type GC, Prognosis, Chemotherapy, Immune checkpoint inhibitor

## Abstract

**Background:**

Although recent advances in high-throughput technology have provided many insights into gastric cancer (GC), few reliable biomarkers for diffuse-type GC have been identified. Here, we aim to identify a prognostic and predictive signature of diffuse-type GC heterogeneity.

**Methods:**

We analyzed RNA-seq-based transcriptome data to identify a molecular signature in 150 gastric tissue samples including 107 diffuse-type GCs. The predictive value of the signature was verified using other diffuse-type GC samples in three independent cohorts (*n* = 466). Log-rank and Cox regression analyses were used to estimate the association between the signature and prognosis. The signature was also characterized by somatic variant analyses and tissue microarray analysis between diffuse-type GC subtypes.

**Results:**

Transcriptomic profiling of RNA-seq data identified a signature which revealed distinct subtypes of diffuse-type GC: the intestinal-like (INT) and core diffuse-type (COD) subtypes. The signature showed high predictability and independent clinical utility in diffuse-type GC prognosis in other patient cohorts (HR 2.058, 95% CI 1.53–2.77, *P* = 1.76 × 10^–6^). Integrative mutational and gene expression analyses demonstrated that the COD subtype was responsive to chemotherapy, whereas the INT subtype was responsive to immunotherapy with an immune checkpoint inhibitor (ICI). Tissue microarray analysis showed the practical utility of IGF1 and NXPE2 for predicting diffuse-type GC heterogeneity.

**Conclusions:**

We present a molecular signature that can identify diffuse-type GC patients who display different clinical behaviors as well as responses to chemotherapy or ICI treatment.

**Electronic supplementary material:**

The online version of this article (10.1007/s10120-019-01029-4) contains supplementary material, which is available to authorized users.

## Introduction

Gastric cancer (GC) is the third leading cause of cancer-related mortality and the fifth most common cancer worldwide [1]. Chemotherapy has been well established to improve the survival rates of GC patients after surgery [2]. Even with the advancement of therapeutic options, however, the optimal approach for an individual GC patient is difficult to determine [2] because of considerable clinicopathologic heterogeneity in GC patients. The Lauren classification, stratifying GC into diffuse, intestinal, and mixed types, has been widely used in the clinical field [3, 4]. Diffuse-type GC accounts for approximately 30% of GC and often exhibits more aggressive characteristics and poorer clinical outcomes than intestinal-type GC [5, 6]. Therefore, there is a crucial need to molecularly characterize diffuse-type GC and identify a signature that could predict the clinical course of and suggest appropriate treatment options for diffuse-type GC.

Among four molecular subtypes [i.e., Epstein–Barr virus positive (EBV), microsatellite instable (MSI), genome stable (GS), and chromosomal instability (CIN)] stratified according to the Cancer Genome Atlas (TCGA) consortium, diffuse-type GC is classified mainly as the GS type [7]. Additionally, diffuse-type GCs can be further distinguished as the microsatellite stable and epithelial-to-mesenchymal transition (MSS/EMT) subtype, which shows the worst prognosis as demonstrated by the Asian Cancer Research Group (ACRG) [8]. Despite these defined classifications, diffuse-type GC is molecularly heterogeneous [5] because of different tumor origins among diffuse-type GCs [9]. Recent advances in molecular characterization have provided evidence that diffuse-type GC comprises molecularly distinct subtypes, including EMT-associated subtypes [4, 10]. Indeed, a number of genome-wide studies have been conducted on diffuse-type GC, yet there are no reliable criteria that can adequately predict prognosis in diffuse-type GC. Moreover, despite the advances in treatment options, including chemotherapy, the ability of these studies to predict the response to therapy remains insufficient, or the relevant cancer patients included were limited.

Here, we investigated distinct molecular subtypes of diffuse-type GC, displaying different prognoses and treatment responsiveness, and generated a gene signature stratifying diffuse-type GC patients into these subtypes. Using multiple patient cohorts, we tested whether our signature showed prognostic or therapeutic relevance. Via integrative exploration of mutational and gene expression alterations, we discovered that high-risk patients classified by the signature benefited from standard chemotherapy, while low-risk patients were responsive to immunotherapy based on immune checkpoint inhibitor (ICI) treatment. Using tissue microarray analysis (TMA) to verify protein expression levels, we also confirmed that IGF1 and NXPE2 might be practical indicators for predicting the heterogeneous clinical behavior of diffuse-type GCs.

## Materials and methods

### Patients and data

We generated a transcriptome dataset of 150 fresh-frozen tissues including diffuse-type GC (*n* = 107), intestinal-type GC (*n* = 23), and normal gastric tissues (*n* = 20) obtained from the BioBank of the Chungnam and Seoul National University Hospitals (the original cohort, *n* = 150). We also obtained the mRNA expression or variant data of diffuse-type GC from the TCGA database (*n* = 61, the TCGA cohort) [7], the ACRG study (*n* = 135, the ACRG cohort) [8], and the Samsung Medical Center in South Korea (*n* = 280, the SMC cohort) [11]. Table S1 details the baseline characteristics of GC patients.

Details of the methodology are available in Supplementary Materials.

## Results

### Discovery of distinct subtypes of diffuse-type GC by transcriptomic profiling

**Fig. 1:**
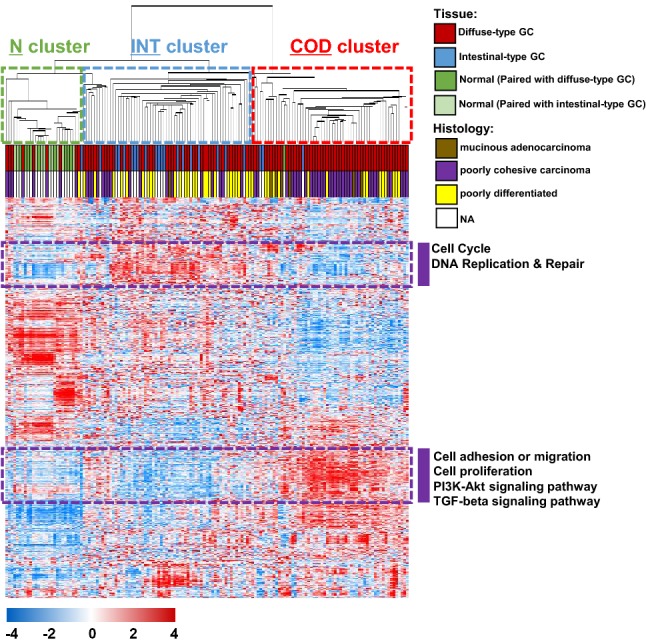
Gene expression patterns of the gastric cancer (GC) samples in the original cohort including diffuse-type tumors (*n* = 150). A total of 3586 genes with showing greater variation in expression changes across the samples were selected for cluster analysis [standard deviation (SD) > 0.9]. The data are presented in matrix format, in which rows represent individual genes, and columns represent each tissue sample. The red and blue colors reflect high and low expression levels, respectively. *NA* not available

We performed gene expression profiling of 150 GC samples in the original cohort. Through unsupervised hierarchical clustering with a total of 3586 genes showing varying expression changes across GC samples [standard deviation (SD) > 0.9], three major sample clusters were observed: the normal-like (N), intestinal-type-like (INT), and core diffuse-type (COD) GC subgroups (Fig. 1). Most normal tissue samples were included in the N cluster (19 out of 20), while the vast majority of intestinal-type GC samples were included in the INT cluster (19 out of 23). Among 59 tissue samples in the COD cluster, 54 samples were diffuse-type GCs. When the histological data were compared among the sample clusters, not surprisingly, patients with poorly cohesive carcinoma (PCC), a poor prognostic histological subtype of GC, were found to be significantly plentiful in the COD cluster (*χ*^2^ test, *P* = 0.002, Fig. 1).

Through exploring the expression patterns, we intuitively identified two distinct subsets of genes that were highly associated with the INT or COD cluster of GC (252 and 397 genes, respectively, surrounded by purple dashed lines in Fig. 1). These genes were more highly expressed in the INT or COD cluster than in the other clusters. Among the 252 genes associated with the INT cluster, many genes involved in the cell cycle or DNA repair were observed. Among the 397 genes associated with the COD cluster, on the other hand, genes involved in EMT-associated functions such as cell adhesion/migration or the TGFβ signaling pathway were significantly plentiful (Fig. 1). These results indicate that the newly discovered COD cluster may be compatible with the known poor prognosis of diffuse-type GC, which may derive from EMT activity.

To identify optimal gene sets for distinguishing patient subgroups (i.e., the N, INT, and COD clusters) in diffuse-type GC, genes that were differentially expressed between the three clusters were next identified using only diffuse-type GC samples (*n* = 107) from the original cohort. We selected two lists of genes that were significantly differentially expressed between the N and INT clusters or the INT and COD clusters (*P* < 0.001), which were compared by using a Venn diagram approach (Fig. S1a). Gene list “A” represents genes that were differentially expressed between the N and INT clusters (737 genes), and gene list “B” represents genes that were differentially expressed between the INT and COD clusters (2069 genes). When the two gene lists were compared, three different patterns were observed: only A (590 genes), A and B (147 genes), and only B (1922 genes; Fig. S1b). Diffuse-type GC samples in the N cluster showed gene expression patterns of normal stomach, which might be due to involving many normal gastric cells, even though all diffuse-type GC tissues used in the current investigation were pathologically confirmed as containing high tumor cell contents. Therefore, genes in the only-A category exhibited distinct expression patterns associated with tumorigenesis of GC in the INT subgroup. On the other hand, genes in the only-B category exhibited expression patterns associated with the progression into the COD subgroup in diffuse-type GC. Genes in both the A and B categories were common to the three subgroups of diffuse-type GC. Among the genes involved in the only-B category, many genes associated with EMT activity, such as *ERG*, *FGF1*, *FGF2*, *FGFR1*, *SFRP1*, *SFRP2*, *SOX10*, *SOX8*, *TGFB1I1*, and *TGFB3*, were highly expressed in the COD subgroup (Fig. S1b), consistent with our previous observations shown in Fig. 1.

### Prognostic utility of the COD signature in diffuse-type GC

**Fig. 2:**
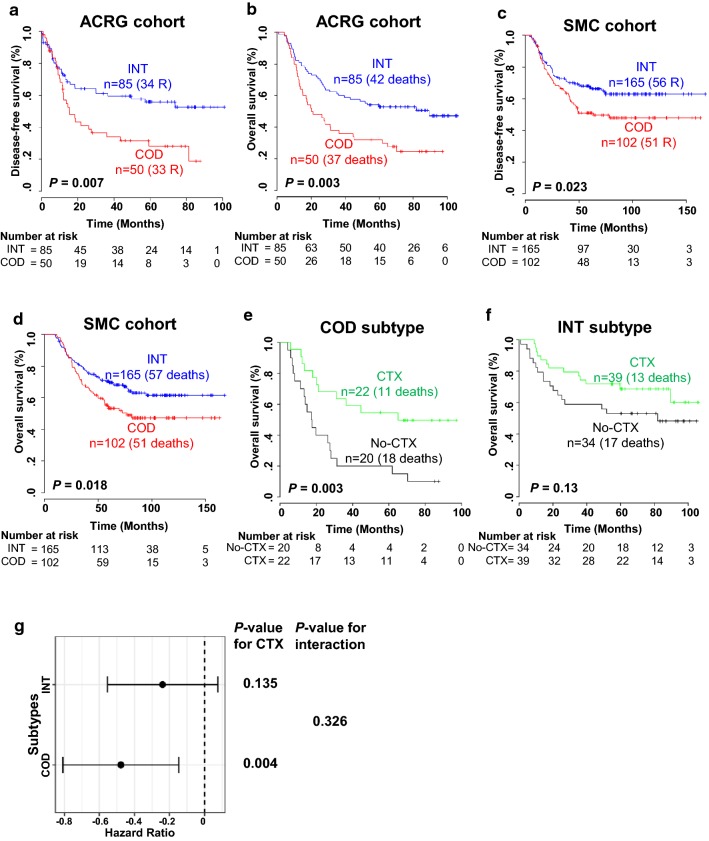
The prognosis of intestinal-like (INT) and core diffuse (COD) subtypes dichotomized by the signature in the multiple gastric cancer (GC) patient cohorts. **a**–**d** Kaplan–Meier curves showing the time to recurrence or death of diffuse-type GC patients in the ACRG and SMC cohorts. *P* values were obtained by log-rank tests. **e**, **f** Kaplan − Meier plots of diffuse-type GC patients of the COD (**e**) and INT (**f**) subtypes. The data were plotted according to whether patients received chemotherapy (CTX) or not. **g** Interaction of the INT and COD subtypes with adjuvant chemotherapy in patients with diffuse-type GC in the ACRG cohort. A Cox proportional hazard model was used to analyze the interaction between the subtypes and adjuvant chemotherapy. The solid line represents the 95% confidence interval of the hazard ratios. *ACRG* Asian Cancer Research Group, *SMC* Samsung Medical Center

**Fig. 3:**
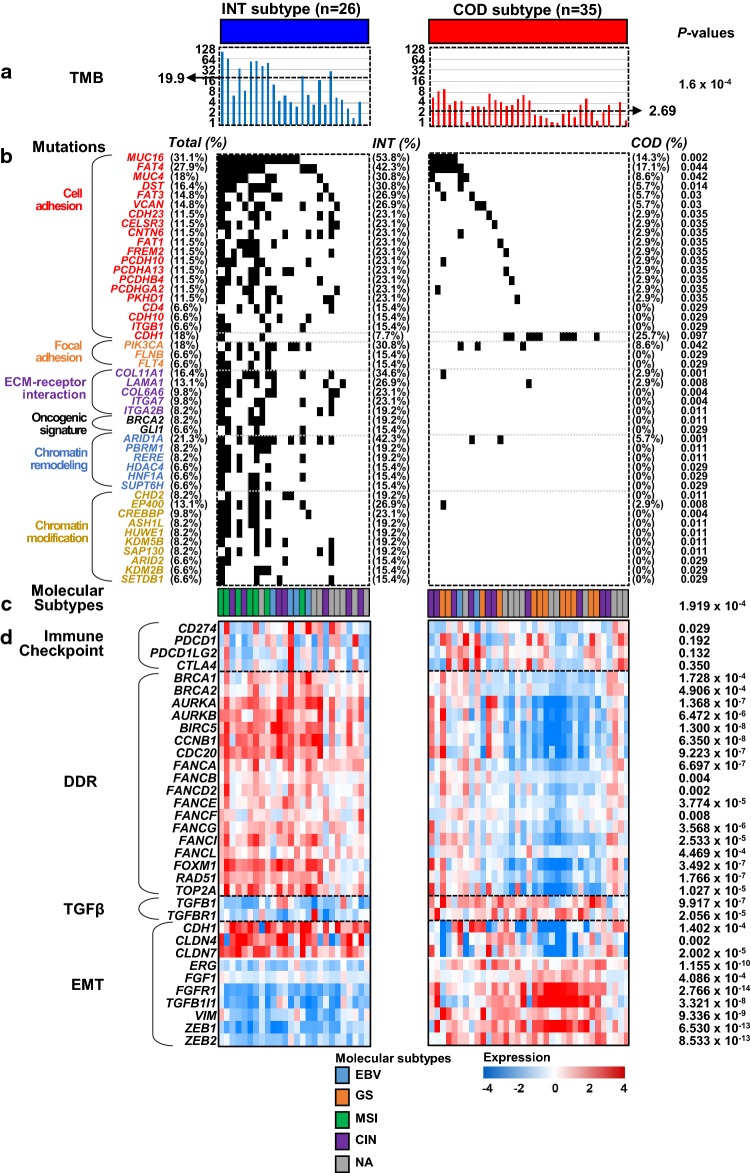
Association between intestinal-like (INT) and core diffuse-type (COD) subtypes and core molecular features in diffuse-type gastric cancer (GC). The molecular characteristics of the two subtypes distinguished by the COD signature were categorized by the tumor mutation burden (TMB) (**a**), mutations (**b**), known molecular subtypes (**c**), and heat maps of the expression of genes involved in core pathways (**d**). In the panel of mutations, gene symbols are subgrouped by a number of enriched functions, such as cell adhesion, focal adhesion, ECM-receptor interaction, the oncogenic signature, chromatin remodeling, and chromatin modification. *P* values in TMB and gene expression categories were obtained by two-sample *t* tests. The *P* value of the molecular subtype was obtained by the *χ*^2^ test, whereas the remaining *P* values of mutations were obtained by Fisher’s exact tests. *DDR* DNA damage response, *EMT* epithelial–mesenchymal transition

We next tried to identify a molecular signature under the influence of 1922 genes in the only-B category and use the signature (also referred to as the COD signature) to classify risk subgroups of diffuse-type GC. On the basis of hierarchical clustering analysis of the expression patterns of these genes in the ACRG cohort (*n* = 135), we obtained two distinct sample clusters (i.e., INT and COD subtypes) based on the expression of genes associated with EMT (Fig. S2a). Regarding the estimated disease-free survival (DFS) of these two patient subgroups, the recurrence rate in the COD subtype was significantly higher than in the INT subtype (log-rank test, *P* = 0.007; Fig. 2a). The overall survival (OS) rate of the COD patients was also significantly lower than that of the INT patients (log-rank test, *P* = 0.003; Fig. 3b), demonstrating prognostic relevance of the COD signature in diffuse-type GC. To validate the prognostic value of the signature, we also used gene expression data for diffuse-type GC from the SMC cohort (*n* = 267). Using the same procedure employed for the ACRG cohort, the patients in the SMC cohort were divided into two subtypes (INT and COD) by hierarchical cluster analysis (Fig. S2b), and the DFS and OS of each group were estimated. Kaplan–Meier analyses revealed that the signature was a significant predictor of diffuse-type GC patient survival in the SMC cohort (log-rank tests,* P* = 0.023 for DFS and *P* = 0.018 for OS; Fig. 2c, d.

To determine the prognostic independence of the signature, we combined the clinical data from two patient cohorts (ACRG and SMC) and applied Cox regression analyses to the signature and known clinicopathological risk factors. In the univariate analysis, the significant prognostic indicators of OS in diffuse-type GC included age and AJCC stage, along with the COD signature (Table 1). When the multivariate test was performed on the combined cohort, the COD signature retained its statistical significance for the OS of diffuse-type GC patients even after applying a variable selection procedure (HR 2.508, 95% CI 1.53–2.766, *P* = 1.76 × 10^–6^; Table 1), illustrating the high prognostic relevance of the signature as an independent risk factor for diffuse-type GC.

**Table 1:** Univariate and multivariate Cox regression analysis of overall survival in diffuse type gastric cancer (combined with ACRG and SMC cohorts)

Variable	Univariate			Multivariate		
	*n*	HR (95% CI)	*P* value	*n*	HR (95% CI)	*P* value
Age	402	1.01 (1–1.03)	0.04	402	1.02 (1.01–1.03)	0.003
Gender (male or female)	402	1.07 (0.81–1.43)	0.625			
AJCC stage (I, II, III or IV)	402	2.52 (2.09–3.03)	3.22 × 10^–22^		2.67 (2.2–3.23)	1.04 × 10^–23^
Tumor site (cardia, body, antrum or whole)	402	0.99 (0.78–1.25)	0.9			
COD-signature (INT or COD^a^)	402	1.68 (1.26–2.23)	4.4 × 10^–4^		2.06 (1.53–2.77)	1.76 × 10^–6^

*ACRG* Asian Cancer Research Group, *SMC* Samsung Medical Center, *HR* hazard ratio, *CI* confidence interval, *INT* intestinal-like, *COD* core diffuse type

^a^Predicted outcome in Fig. 2 was used for analysis (INT or COD subtypes)

Adjuvant chemotherapy data were available for the patients from the ACRG cohort. Because adjuvant chemotherapy is the standard treatment option for GC, we investigated whether the signature could predict diffuse-type GC patients who would benefit from adjuvant chemotherapy. This analysis was performed for patients with diffuse-type GC without distant metastasis (*n* = 115). When the patients were divided into the INT and COD subtypes based on the signature and the difference in OS was independently assessed, adjuvant chemotherapy was found to improve the survival rate in patients with the COD subtype (*P* = 0.003, Fig. 2e), while patients with the INT subtype showed only a moderate benefit from adjuvant chemotherapy (*P* = 0.13, Fig. 2f). When the Cox regression model was applied, the interaction of the signature with adjuvant chemotherapy reached a significance level of 0.326 (Fig. 2g). However, consistent with the Kaplan–Meier and log-rank tests, the estimated HR for adjuvant chemotherapy in the COD subtype was 0.333 (95% CI 0.155–0.713; *P* = 0.004), retaining significant predictive value, while the HR for OS for adjuvant chemotherapy in the INT subtype was 0.576 (95% CI 0.28–1.187; *P* = 0.135). Taken together, the results showed that the newly identified signature exhibited significant prognostic potential as well as predictive value for chemotherapy in diffuse-type GC patients.

### Biological insight into the COD signature

Functional enrichment analysis illustrated that genes involved in cellular movement, interaction, proliferation, or cell morphology in the category of molecular and cellular functions were significantly activated in the COD subtype (Fig. S3). Regulator effects analysis also showed that players involved in the EMT signature were key genetic mediators dichotomizing diffuse-type GC subgroups (Table S2; Fig. S4). Details of biological insights into the COD signature are available in Supplementary Materials.

### Mutational profiling reveals an association between the COD signature and the response to an immune checkpoint inhibitor (ICI)

While the COD signature showed significant predictive value for standard chemotherapy in diffuse-type GC (Fig. 2f–h), patients with the INT subtype responded moderately to this type of therapy, implying a need for additional or alternative therapeutic options for the INT subtype. To identify clues toward alternative therapies for the INT subgroup of patients, we explored mutational variants in diffuse-type GC in the TCGA cohort. A hierarchical cluster analysis based on the signature revealed stratification of patients into the INT and COD subtypes (Fig. S5). Estimation of the tumor mutation burden (TMB) in the INT and COD subtypes revealed that TMBs in the INT subtype were significantly higher than those in the COD subtype (Two-sample *t* test; *P* = 1.6 × 10^–4^; Fig. 3a). Comparison of the mutation frequencies of all known genes between the INT and COD subtypes revealed that a total of 470 genes showed significantly different mutation frequencies (Fisher’s exact tests, each *P* < 0.05; Table S3). We searched for enriched functions using significant mutational variants (Fig. S6) and observed that genes involved in cell/focal adhesion, ECM-receptor interaction, or chromatin remodeling/modification were significantly plentiful (Fig. 3b), consistent with our previous results (Fig. 1; Fig. S3). Among the genes involved in cell adhesion, *MUC16*, associated with hypermutation and favorable prognosis in GC [12], and with resistance to chemotherapy in lung cancer [13], was the top discriminator of the INT and COD subtypes. The total mutation rate of *MUC16* in the diffuse-type GC patients was 31.1%, and the mutation frequency of *MUC16* in the COD subtype was significantly higher than that in the INT subtype (Fisher exact test; *P* = 0.002), suggesting that the *MUC16* variant is a good indicator discriminating diffuse-type GC of the INT subtype from that of the COD subtype. Comparison of *CDH1* mutations, which are well-known variants in diffuse-type GC, between the INT and COD subtypes revealed that the mutation frequency in the COD group was higher than that in the INT group; however, the difference was not statistically significant. We also observed significantly more mutations of *PIK3CA* [frequently found in microsatellite unstable (MSI) GCs] [14] and *ARID1A* (associated with MSI along with hypermutations and PD-L1 expression in cancers including GC) [15–17] in the INT subtype compared to the COD subtype. These results suggest distinct features of the INT subtype, including high TMB and MSI, which are typical indicators predicting the response to ICI treatment [5, 18, 19].

We also compared known molecular subtypes [7] with somatic alterations illustrated by the signature (Fig. 3c). When considering molecular subtypes, all MSI (*n* = 7) and the majority of EBV (4 out of 6) patients exhibited the INT subtype, whereas all GS (*n* = 12) patients were classified as the COD subtype. The difference in molecular subtypes between the two subgroups of the signature was statistically significant (*χ*^2^ test; *P* = 1.919 × 10^–4^; Fig. 3c). These results also support a distinct feature of the INT subtype regarding the response to ICI treatment according to significant enrichment of MSI or EBV along with *PIK3CA* and *ARID1A* mutations [5, 14–17].

We further sought to identify the predictive value of the COD signature for ICI treatment (Fig. 3d). When the expression levels of immune checkpoint genes were compared, *CD274* (*PD-L1*) showed a significant difference in expression between the INT and COD subtypes, supporting a reported close relationship between *ARID1A* and *PD-L1* expression in GC [15–17]. Since activation of DNA damage response and repair (DDR) genes is significantly associated with the response to ICI [20], we also estimated the expression levels of DDR genes, revealing that the vast majority of DDR genes were significantly activated in the INT subtype. When the expression levels of members of the TGFβ pathway and its associated factors in EMT were estimated, it was found that TGFβ pathway genes were significantly downregulated in the INT subtype, and EMT genes were differentially expressed between the two subtypes, consistent with the previous report that TGFβ attenuates the response to ICI [21]. Because a dataset of ICI responsiveness in GC is publicly available, we sought to validate the predictive value of the COD signature for ICI treatment [22]. After applying the signature to the transcriptome data from GC patients and dividing them into the INT and COD subtypes, it was found that the rate of ICI responsiveness was significantly higher in the INT group than the COD group (Fig. S7), indicating a possible treatment option using ICI in patients classified into the INT subtype. Considering these findings together with gene expression and mutation profiling results, we suggest that the gene expression-based COD signature reflects ICI responsiveness. However, we also indicate that more rigorous validation steps are needed, because of insufficient GC samples availability.

To more characterize the INT and COD subtypes at copy number or epigenetic alterations, we also performed copy number variation (CNV) and methylation profiling in the TCGA cohort. When exploring CNV data, we observed 842 genes had statistically significant difference in CNV between diffuse-type patients with the INT and COD subgroups. A number of important canonical pathways associated with these genes including cell adhesion molecules were found to be enriched (Fig. S8). When a methylation profiling was carried out for comparing epigenetic alterations between two subtypes, 1412 genes had statistically significant differences in methylation between the INT and COD subtypes. A function enrichment test using these genes revealed that many important canonical pathways including focal or cell adhesion, cell adhesion molecules, and ECM-receptor interaction were found to be enriched (Fig. S9), consistent with the results of gene expression and mutation profiling.

### Practical utility of the IGF1 and NXPE2 proteins for classifying diffuse-type GC of the INT and COD subtypes

**Fig. 4:**
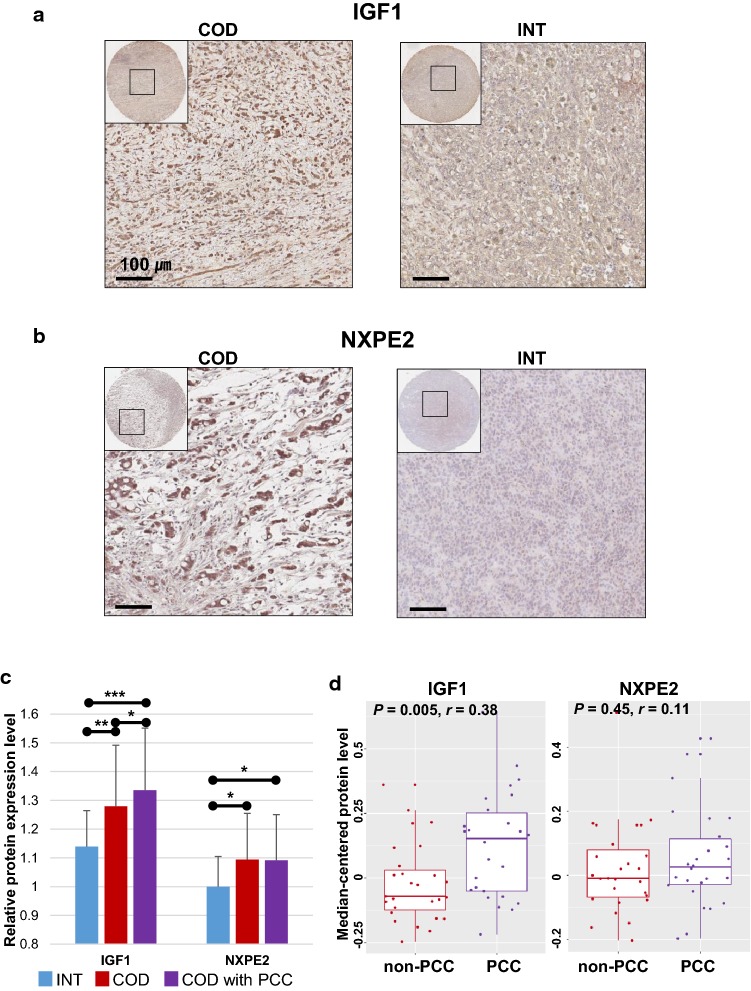
Confirmation of the expression levels of the IGF1 and NXPE2 proteins in patients with diffuse-type gastric cancer (GC). **a** Comparison of images of IGF1 protein expression generated via tissue microarray analysis between diffuse-type patients with the core diffuse (COD) and intestinal-like (INT) subtypes. **b** Comparison of images of NXPE2 protein expression generated via tissue microarray analysis between diffuse-type patients with the COD and INT subtypes. **c** Comparative analysis of the protein expression levels of IGF1 and NXPE2 between diffuse-type GC patients with the INT, COD, and COD with poorly cohesive carcinoma (PCC) subtypes. Each bar represents the mean ± standard deviation of three independent experiments. **d** Comparison of the expression levels of the IGF1 and NXPE2 proteins between the histological non-PCC and PCC subclasses. IGF1 protein expression levels showed a statistically significant correlation with the histological subtypes. *r* values were obtained by biserial correlation tests and *P* values were obtained by two-sample *t* tests. *P < 0.05, **P < 0.01, ***P < 0.001

We sought to identify the best candidates that could practically discriminate GC patients of the COD subtype from those of the INT subtype. Among upstream regulatory candidates (Table S2), we selected *IGF1* as a good indicator distinguishing the two subgroups, showing the greatest fold difference in mRNA expression (5.04-fold) between the INT and COD subtypes. We also selected *NXPE2* as a novel indicator whose relationship with GC has not yet been described. *NXPE2* exhibited the greatest fold difference in mRNA expression (29.1-fold) between the INT and COD subtypes among the novel genes. With these two candidates, we carried out protein expression analysis using a tissue microarray, revealing that these proteins were more highly expressed in tumor cells with the COD subtype than in those with INT subtype (Fig. 4a, b). When protein expression levels were compared between subtypes, IGF1 and NXPE2 were found to show significantly higher expression in the COD subtype than in the INT subtype. Further stratification into COD subtype GC patients with histological PCC revealed significantly higher expression of the IGF1 protein than in the other subtypes, whereas no such significant elevation of NXPE2 expression was observed (Fig. 4c), suggesting IGF1 as a good indicator correlated with molecular as well as histological subtypes. By estimating the correlations between histological subtypes and protein expression levels, we found that IGF1 expression was significantly associated with histological PCC, while NXPE2 did not show a significant relationship with the histological classification (Fig. 4d). These results suggest that IGF1 may be a good clinical indicator for classifying high-risk diffuse-type GC patients and supporting histological subclassification systems.

### NXPE2 promotes cell migration and proliferation in vitro

Based on our new finding of NXPE2 characteristics, we sought to verify the effects of cell migration and proliferation of NXPE2 in diffuse-type GC cell lines. We ectopically overexpressed NXPE2 in the SNU601 and MKN45 cells, and the expression was successfully overexpressed in two cell lines using RT-PCR (Fig. S10a). Then, we performed migration assays with NXPE2 overexpressing GC and control cells. We found that ectopic NXPE2 overexpression significantly increased the migration ability in SNU601 and MKN45 cells (Two sample *t* tests; *P* = 1.48 × 10^–6^ and *P* = 0.019 in SNU601 and MKN45, respectively; Fig. S10b). We also performed cell proliferation assays with ectopic NXPE2 overexpressing GC and control cells. Although moderate proliferation of SNU601 cells was observed (at 48 h), we found that ectopic NXPE2 overexpression significantly increased the proliferation of MKN45 cells (Fig. S10c). These results suggest that NXPE2 mediates diffuse-type GC aggressiveness promoting cell migration or proliferation, which are key determinants of malignant progression and metastasis.

## Discussion

Diffuse-type GC is clinically heterogeneous and frequently exhibits extremely poor outcomes [5]. Using multiple GC patient cohorts, we carried out transcriptome and mutation profiling analyses, which identified a signature of distinct prognostic subtypes of diffuse-type GC. The COD signature showed significant prognostic relevance with independent utility in relation to other pathological factors. The signature also showed therapeutic relevance in that patients with the COD subtype benefit from standard chemotherapy, while patients with the INT subtype are responsive to ICI treatment. Additionally, tissue microarray analyses revealed that IGF1 and NXPE2 might be useful for predicting different clinical behaviors of diffuse-type GC (Fig. S11).

Considerable efforts have been devoted to elucidating the molecular characteristics and establishing prognostic models of GC [4, 5, 7, 8, 10, 23, 24]. Recent advanced investigations characterizing diffuse-type GC at the proteomics level demonstrate the practical utility of specific proteins in addressing aggressive diffuse-type GC in the clinical field [4, 5, 10]. Despite these contributions, the ability to predict the clinical course of patients with diffuse-type GC remains a major clinical challenge. Through our effort to generate new transcriptome data from GC patients involving more than 100 diffuse-type GCs, we identified a molecular signature for classifying distinct prognostic subtypes of diffuse-type GC. The patients with diffuse-type GC classified as exhibiting the COD subtype potentially benefited from chemotherapy, whereas those of the INT subtype might be responsive to immunotherapy with ICI. These data underscore the importance of the molecular subtypes defined by the COD signature as a potential prognostic and predictive signature in diffuse-type GC.

Using recently updated data from the TCGA consortium, the current study revealed two distinct molecular subtypes of diffuse-type GC and several molecular features responsible for their activity. The subgroups of diffuse-type GCs showed different molecular characteristics between the INT and COD subtypes, where INT subtype included many MSI and EBV patients, whereas the COD subtype mainly included GS patients. Genes involved in the DDR were significantly activated in the INT subtype, while many EMT genes were highly activated in the COD subtype. While the response rate to standard chemotherapy in the COD subtype was significantly high, no such responsiveness was observed in the INT subtype, implying a crucial need for alternative treatment options in diffuse-type GC patients classified into the INT subtype. Through integrative gene expression and mutational analysis of diffuse-type GC, we discovered a number of distinct molecular features of the INT subtype, such as high TMB, enrichment of MSI or EBV molecular subtypes, activation of DDR genes, and inactivation of the TGFB1 pathway along with its downstream effectors related to EMT activity, indicating favorable responsiveness to ICI treatment in the INT subtype. The considerable molecular difference between the COD and INT subtypes of diffuse-type GC supports the practical utility of the COD signature in determining the clinical behavior and treatment options of diffuse-type GC patients. However, because of limited data availability in GC associated with treatment responsiveness, further validations are needed.

We also verified two proteins, IGF1 and NXPE2, as practical indicators for predicting the clinical course of diffuse-type GC. IGF1, insulin-like growth factor 1, is similar to insulin in its function and structure and is a member of a family of proteins involved in mediating growth and development. IGF1 is involved in signaling cross-talk at multiple levels with various components of the TGFβ signaling pathway, and its activity is associated with the activation of Akt, which increases cell survival, proliferation, and malignant transformation [25], consistent with our observations (Fig. 1; Fig. S4). IGF1 is also known as an indicator of a mesenchymal phenotype in GC [24], which was identified as a corresponding molecular feature of the COD subtype in the current investigation. When associations with pathological criteria were estimated, IGF1 was found to present a significant correlation with PCC, a histological subtype of GC with a poor prognosis (Fig. 4d). These results suggest that IGF1 is a good indicator for selecting high-risk diffuse-type GC patients. NXPE2, neurexophilin and PC-esterase domain family member 2, was also surveyed as a new predictive indicator. While several associations of the NXPE2 protein with inflammatory diseases such as Crohn’s disease [26], ulcerative colitis [27], and inflammatory bowel disease [28] have recently been reported, we identified discriminatory ability of the NXPE2 protein in classifying two distinct subtypes of diffuse-type GC, suggesting NXPE2 as a novel indicator predicting high-risk diffuse-type GC patients. Since no association with GC or druggable compounds has yet been described, more rigorous efforts to characterize NXPE2 are urgently needed.

In conclusion, we identified a signature distinguishing diffuse-type GC into molecular subtypes exhibiting different prognostic characteristics. Our results also confirmed a chemo-sensitivity and an ICI responsiveness of the molecular subtypes classified by the signature. Although our data demonstrate that the signature have significant prognostic and predictive values, a further validation study is needed to identify a limited number of biomarkers that still retain the robustness of our signature.

## Electronic supplementary material

Below is the link to the electronic supplementary material.

Supplementary file1 (PDF 1956 kb)
