# Supplementary material for: Identification of a molecular signature of prognostic subtypes in diffuse-type gastric cancer
Source: Gastric Cancer. 2019 Nov 26;23(3):473–82. doi: 10.1007/s10120-019-01029-4 (PMC7165151; doi:10.1007/s10120-019-01029-4)
Supplement: Supplementary file 1 — Supplementary file1 (PDF 1956 kb) [file 10120_2019_1029_MOESM1_ESM.pdf]

Journal: Gastric Cancer

Identification of a molecular signature of prognostic subtypes in diffuse-type gastric cancer

Seon-Kyu Kim[1†], Hee-Jin Kim[2†], Jong-Lyul Park[2], Haejeong Heo[1,3], Seon-Young Kim[1,3], Sang-Il Lee[4], Kyu-Sang Song[5], Woo-Ho Kim[6], and Yong Sung Kim[2,3*]

[1]Personalized Genomic Medicine Research Center, Korea Research Institute of Bioscience and Biotechnology, Daejeon 34141, Korea; [2]Genome Editing Research Center, KRIBB, Daejeon 34141, Korea; [3]Department of Bioscience, University of Science and Technology, Daejeon 34113, Korea; [4]Department of General Surgery, College of Medicine, Chungnam National University, Daejeon 35015, Korea; [5]Department of Pathology, College of Medicine, Chungnam National University, Daejeon 35015, Korea; [6]Department of Pathology, Faculty of Medicine, Seoul National University, Seoul 03080, Korea.

[†]Seon-Kyu Kim and Hee-Jin Kim contributed equally.

[*]Correspondence:

Dr. Yong Sung Kim; Genome Editing Research Center, Korea Research Institute of Bioscience and Biotechnology, 125 Gwahak-ro, Yuseong-gu, Daejeon 34141, Korea; E-mail: yongsung@kribb.re.kr; Tel: +82-42-879-8110; Fax: +82-42-879-8119

Supplementary methods

Patients and tissue samples

Fresh-frozen gastric tissues (n = 150) were obtained from the BioBank of the Chungnam and Seoul National University hospitals. Notably, all tissues were not randomly but manually selected for collecting more than 100 diffuse-type gastric (GC) tissues to identify molecular subtypes in diffuse-type GC. Consequently, a total of 150 fresh-frozen tissues including diffuse-type GC (n = 107), intestinal-type GC (n = 23), and normal gastric tissues (n = 20) were obtained (the original cohort, n = 150). All normal gastric samples were obtained from surrounding normal tissues of tumors, among which 10 samples were paired with diffuse-type GCs (n = 10) while other 10 samples were paired with intestinal-type GCs (n = 10). Because diffuse-type GC tissues are mixed with various types of cells, we selected tissues containing a high tumor cell content (>80%) by histopathologic assessment. The collection and analysis of all samples was approved by the Institutional Review Board of Chungnam and Seoul National University hospitals, and informed consent was obtained from each subject. The baseline characteristics of the GC patients in the original cohort are described in Table S1.

RNA extraction, RNA-seq experiments and data processing

Total RNA was isolated using the RNeasy Mini Kit (Qiagen, CA, USA) according to the manufacturer's protocol. The quality and integrity of the RNA were confirmed by agarose gel electrophoresis and ethidium bromide staining, followed by visual examination under ultraviolet light. The sequencing library was prepared using the TruSeq RNA Sample Preparation kit v2 (Illumina, CA, USA) according to the manufacturer's instructions. In brief, mRNA was purified from total RNA using poly-T oligo-attached magnetic beads, fragmented, and converted into cDNA. Adapters were then ligated to the cDNA, and the fragments were

amplified by PCR. Sequencing of paired-end reads (2×75 bp) was performed using the NextSeq 500 platform (Illumina).

Reference genome sequence data from *Homo sapiens* were obtained from the University of California, Santa Cruz, Genome Browser Gateway (assembly ID: hg38). The reference genome indexing and read mapping of tissue samples were performed using STAR software (ver. 2.5.3a). The dataset generated by RNA-seq is available in the National Center for Biotechnology Information (NCBI) Gene Expression Omnibus (GEO) public database under data series accession number GSE113255.

Public datasets of GC patients

To verify the prognostic value of the signature identified in the original cohort, a gene expression dataset from GC patients from the Asian Cancer Research Group (ACRG) study was used [1]. Among the microarray profiles from 300 GCs in this cohort, 135 samples of diffuse-type GC were used as the first validation dataset (the ACRG cohort, GSE62254, n = 135). In the ACRG cohort, 62 patients with diffuse-type GC received postoperative chemotherapy (either single-agent 5-fluorouracil or a combination of 5-fluorouracil and cisplatin/oxaliplatin, doxorubicin, or paclitaxel). We also used another gene expression dataset of GC patients that was generated by the cDNA-mediated Annealing, Selection, extension and Ligation (DASL) assays retrieved from the Samsung Medical Center in South Korea [2]. Among the 432 GC patients in the cohort, gene expression data from 267 diffuse-type GCs were used as the second validation dataset (the SMC cohort, GSE26253, n = 267). Data on adjuvant chemotherapy were not available in the SMC cohort. RNA-seq, exome-seq, copy number variation (CNV), and methylation data provided by the Cancer Genome Atlas (TCGA) consortium were also used to explore gene expression, mutational, copy number, and epigenetic alterations [3]. Among the GC samples in the TCGA data, we only included

61 diffuse-type GCs (TCGA cohort, n = 61). Notably, the mRNA quantification and DNA variant data with clinical information on GC for the TCGA cohort were obtained from the GDC portal (https://portal.gdc.cancer.gov/), in which tumor histology was last updated in March 2017. CNV and methylation data of 61 diffuse-type GC patients were obtained from the cBioPortal (https://www.cbioportal.org/). The baseline characteristics of public datasets of the GC patients along with our original cohort are described in the Table S1.

Statistical analysis

To perform gene expression profiling for gastric tissue samples, a hierarchical clustering algorithm with the centered correlation coefficient as the measure of similarity and complete linkage clustering was applied. For cluster analysis, the read counts per million fragments mapped (CPM) of each sample were used to estimate the expression level of each gene. The CPM data were normalized by the quantile method, log_2 transformed, and median centered across genes and samples.

To estimate the significance of differences in gene expression between sample subgroups, the edgeR package, which uses a negative binomial model was employed to detect differentially expressed genes from count data [4]. Expression differences in genes were considered statistically significant if the *P*-value was <0.001 and the fold difference in expression between two sample groups was ≥2. The Kaplan−Meier method was used to calculate disease-free and overall survival, and the difference in survival between two groups was assessed using log-rank statistics. The prognostic association between the signature and known clinico-pathological factors was assessed using multivariate Cox proportional hazard regression models. A backward-forward step procedure was applied to optimize the multivariate model with the most informative variables [5]. Statistical analysis was mainly carried out in the R language environment (ver. 3.5.3).

Gene set enrichment analysis was carried out to identify the most significant gene sets associated with disease processes, molecular and cellular functions, and physiological and developmental processes. The significance of overrepresented gene sets was estimated by Fisher's exact test. To identify predominant upstream regulators that account for the observed gene expression changes, an upstream regulator analysis was carried out. This analysis determined the number of known targets of each regulator and compared their direction of change to what is expected from the previous literature. An overlap *P*-value and an activation *Z*-score for each potential regulator were estimated. The overlap *P*-value, estimated by Fisher's exact test, measures whether there is statistically significant overlap between the genes in a dataset and the genes regulated by a regulator. The activation *Z*-score is used to infer likely activation states of regulator candidates by comparison with a model that assigns a random regulation direction. A positive or negative activation *Z*-score implies that a potential upstream regulator is activated or inhibited, respectively. Gene set enrichment and upstream regulator analyses were performed using the Ingenuity Pathway Analysis (IPA) tool.

For comparing relative linear CNV data for each gene generated from the Affymetrix SNP6 platform in the TCGA cohort, a two-sample t-test was used. When comparing β-values for each gene generated from the Illumina 450K array platform in the TCGA cohort, a Wilcoxon rank sum test was applied.

Tissue microarray analysis

To examine the protein expression levels of IGF1 and NXPE2, TMA blocks were assembled using 2 mm-diameter core tissue samples from 81 individual paraffin-embedded gastric tumors (including 53 diffuse-type GCs matching RNA-seq data) resected at Seoul National University Hospital. The slides from the TMA blocks were subjected to multiplex

immunohistochemistry (IHC) with three antibodies, targeting IGF-1 (Cat. No. NBP2-48922, NOVUS, Abingdon, UK), NXPE2 (FAM55B, NBP2-31001, NOVUS, Abingdon, UK), and cytokeratin (CK, Cat. No. M3515, Agilent). The first IHC assay was performed with anti-IGF-1, followed by the corresponding secondary antibody (rabbit anti-mouse IgG; Invitrogen, Waltham, MA, USA) and an anti-rabbit Envision+ System horseradish peroxidase-labelled polymer. The slides were treated with ImmPact NovaRED (Vector Laboratories, Burlingame, CA, USA) followed by Mayer's hematoxylin. After obtaining scanned images of the whole slides with an AT2 scanner (Leica Biosystems), the slides were treated with stripping buffer (20% SDS, 0.5 M Tris-HCl pH 6.8, β-mercaptoethanol and DW). The slides were then stained with anti-NSPE2, and the above staining-scanning-stripping procedures were repeated. Finally, the slides were stained with anti-cytokeratin, and the images were scanned. For image analysis, each file of a scanned whole slide was divided into small images comprising single cores as the unit of analysis. After the alignment of three images from IHC, tumour cells were identified using CK images and nuclear images. The ratio of the staining intensity of IGF-1 or NXPE2 to that of cytokeratin was measured in all tumor cells.

Human NXPE2 expression vector cloning and establishment of stable cell lines with lentivirus infection

For NXPE2 expression vector cloning, the full-length *NXPE2* cDNA was isolated from a human GC cell line using PCR with the forward primer: 5'-ATATTCTAGAGCCACCATGGTGGAGAAAATACTCATCCAT-3' and reverse primer: 5'-ATATGCGGCCGCCTAACAAATGTAGTTTAAGAACATGCC-3'. The PCR product digested with *Xba*I and *Not*I was cloned pCDH-CMV-MCS-EF1-Puro (System Biosciences). To confirm that the vector contained a wild-type gene without any mutations, the construct was sequenced in both directions. For lentivirus construction, HEK293T cells were seeded at

density of $3x10^5$ cells in 6-well plates and then cultured for 24 hours before transfection, and then co-transfected with the NXPE2 expression vector and the MISSION Lentiviral Packaging Mix (Sigma Aldrich, St. Louis, MO) using Lipofectamine 2000 (Thermo Fisher). Viral particles were collected in soup. DGC cell lines, SNU601 and MKN45 cells, were infected and selected for 7 days with 1 ug/mL puromycin (Sigma Aldrich, St. Louis, MO). Surviving cells were collected for further study. The *NXPE2* mRNA expression was examined with RT-PCR.

RT-PCR and real-time quantitative RT-PCR

RT-PCR and qRT-PCR analyses were performed to validate the overexpression of *NXPE2* in stable cell lines. Total RNA (1 ug) was reverse-transcribed with Superscript II reverse transcriptase (Invitrogen, Carlsbad, CA) for 1 hour at 42℃ and for 5 minutes at 85℃. The primer sequences for RT-PCR were forward primer, 5'-AGACTCCTTTCCCCAGTGGT-3' and reverse primer, 5'-CCAGCTCCATGATGATCAAA-3'. The PCR condition were as follows: 94℃ for 5 minutes followed by 33 cycles at 94℃ for 30 seconds, at 58℃ for 30 seconds, and 72℃ for 30 seconds and a final cycle of 72℃ for 7 minutes. The RT-PCR products (208 bp) were analyzed on 1.5% agarose gels and each sample was compared after adjustment for *ACTB* gene as a control. The reverse transcribed product (100ng) was amplified by 45 cycles with 2x SYBR Green Supermix (Bio-Rad, Hercules, CA) using the primer set Real-time PCR was performed in a C1000 Thermal Cycler (Bio-Rad, Hercules, CA, USA). The *ACTB* gene was amplified as a control. Relative quantification of target mRNA was analyzed by comparative threshold cycle (Ct) methods.

Cell migration and cell viability assay

For migration assay, transwell membrane (Corning) were spread with 0.25 mg/mL of fibronectin (Sigma Aldrich) and dried for 30 minutes. Transwell were inserted into 600 ul of media with 10% FBS on 24-well plate. SNU601 and MKN45 cells seeded at a density of $1x10^5$ cells/well with serum-free media into transwell. After 24 hours of incubation, cells passed membrane were stained with crystal violet and counted. All migration experiments were performed in triplicate. For proliferation assay, the transfectant cells of NXPE2 were plated onto a 96-well plate at a density of $1x10^3$ cells/well and the proliferation over a given time course was measured with the EZ-Cytox Cell Viability Assay Kit (ITSBIO, Seoul, Korea) using a microplate reader (Molecular Devices) at a 450 mm. All cell viability assay was performed in triplicate. Statistical analysis was performed using Student's t-test and a *P*-value <0.05 was considered significant.

Supplementary results

Biological insight into the core diffuse-type (COD) signature

To characterize the biological relevance of the signature, we performed a gene set enrichment test of the 1,922 genes in the signature (Fig. S1). The test illustrated significantly altered functions (Fig. S3), among which many genes involved in cellular movement, cell-to-cell signaling and interaction, cellular growth and proliferation, or cell morphology in the category of molecular and cellular functions were significantly enriched, accounting for the poor prognosis of the COD subtype.

To explore the predominant regulators and their biological activities in the COD subtype, an upstream regulator analysis of 1,922 genes in the signature was carried out. This analysis illustrated many important activated or inhibited regulators, among which several genes involved in the epithelial-to-mesenchymal transition (EMT) signature, such as *TGFB1*, *SNAI1* and *TWIST1,* were significantly activated in the COD subtype. In addition, many regulators playing key roles in cell proliferation, such as *GLI1*, *MYOCD* or *CCL2*, were strongly activated along with the top regulator *TGFB1* (Table S2), indicating that an interactive regulatory network consisting of several regulators may be a key genetic determinant associated with the poor prognosis of diffuse-type GC in COD patients.

Based on the identification of upstream regulators, we performed an analysis of the top regulatory effects indicated by the IPA tool to assess which biofunctions were significantly activated by upstream regulators and their effectors revealed in the upstream regulator analysis. Exploration of the regulatory effects of COD regulators (Table S2) revealed a network of top regulatory effects activating the migration of cells and cell movement of tumor cells (Fig. S4). Upstream regulators such as *TGFB1, SNAI1, GLI1, HDAC3,* and *HDAC6* regulated the activity of many downstream effectors, including *TGFB1I1* and *FGF1*, that are involved in the EMT signature, consistent with our previous findings (Fig. 1; Fig.

S1). We also observed that active *TGFB1* regulated *IGF1*, an indicator of GC with a mesenchymal phenotype [6], and subsequently activated the movement of tumor cells. These results demonstrated a robust property of the COD signature reflecting EMT activity, in which important regulators synergistically activate downstream effectors, leading to cell movement or migration disorders.

References

1. Cristescu R, Lee J, Nebozhyn M, Kim KM, Ting JC, Wong SS, et al. Molecular analysis of gastric cancer identifies subtypes associated with distinct clinical outcomes. Nat Med. 2015;21:449-56.
2. Lee J, Sohn I, Do IG, Kim KM, Park SH, Park JO, et al. Nanostring-based multigene assay to predict recurrence for gastric cancer patients after surgery. PLoS One. 2014;9:e90133.
3. Cancer Genome Atlas Research N. Comprehensive molecular characterization of gastric adenocarcinoma. Nature. 2014;513:202-9.
4. Robinson MD, McCarthy DJ, Smyth GK. edgeR: a Bioconductor package for differential expression analysis of digital gene expression data. Bioinformatics. 2010;26:139-40.
5. Venables WN, Ripley BD, Venables WN. Modern applied statistics with S. 4th ed. New York: Springer; 2002. xi, 495 p. p.
6. Oh SC, Sohn BH, Cheong JH, Kim SB, Lee JE, Park KC, et al. Clinical and genomic landscape of gastric cancer with a mesenchymal phenotype. Nat Commun. 2018;9:1777.

Supplementary tables

Table S1. Baseline characteristics of gastric cancer patient cohorts.

Variables	Original cohort	ACRG cohort	SMC cohort	TCGA cohort
Patients (n)	130	300	432	375
Age (years)				
Median	60	64	53	74
Range	36-85	24-86	23-74	39-98
Gender (%)				
Male	81 (62.3)	199 (66.3)	280 (64.8)	241 (64.2)
Female	49 (37.7)	101 (33.7)	152 (35.2)	134 (35.7)
Location of tumour (%)				
Antrum	57 (43.8)	155 (51.7)	0 (0)	133 (35.5)
Boby	51 (39.2)	107 (35.7)	361 (83.6)	90 (24)
Cardia	7 (5.4)	32 (10.7)	53 (12.3)	91 (24.3)
Whole	2 (1.5)	6 (2)	17 (3.9)	16 (4.3)
Others	13 (10)	0 (0)	1 (0.2)	45 (12)
Grade (%)				
W/D~M/D tubular	0 (0)	123 (41)	111 (25.7)	
P/D tubular	57 (43.8)	116 (38.7)	200 (46.3)	
Signet ring cell	58 (44.6)	37 (12.3)	101 (23.4)	13 (3.5)
Mucinous	7 (5.4)	8 (2.7)	14 (3.2)	19 (5.1)
Others	8 (6.2)	16 (5.3)	6 (1.4)	
NA				343 (91.4)
Lauren type				
Intestinal	23 (17.7)	146 (48.7)	139 (32.2)	71 (18.9)
Diffuse	107 (82.3)	135 (45)	280 (64.8)	61 (16.2)
Mixed	0 (0)	19 (6.3)	13 (3)	1 (0.3)
NA				242 (64.5)
T classification				
T1	7 (5.4)	0 (0)	38(8.8)	
T2	12 (9.2)	186 (62)	317 (73.4)	
T3	30 (23.1)	91 (30.3)	77 (17.8)	
T4	81 (62.3)	21 (7)	0 (0)	
NA				375 (100)
N classification				
N0	21 (16.2)	38 (12.7)	40 (9.3)	
N1	20 (15.4)	131 (43.7)	223 (51.6)	
N2	24 (18.5)	80 (26.7)	103 (23.8)	
N3	65 (50)	51 (17)	66 (15.3)	
NA				375 (100)
AJCC stage				
I	9 (6.9)	30 (6.9)	68 (15.7)	53 (14.1)
II	25 (19.2)	97 (22.5)	167 (38.7)	111 (29.6)
III	86 (66.2)	96 (22.2)	130 (30.1)	150 (40)
IV	10 (7.7)	77 (17.8)	67 (15.5)	38 (10.1)
NA				23 (6.1)
Adjuvant chemotherapy				
Yes		144 (48)		
No		156 (52)		
NA	130 (100)		432 (100)	375 (100)
Death, n	NA	152	167	149
Median follow-up (month)	NA	57.9	61.9	14.7

Abbreviation: ACRG, Asian Cancer Research Group; SMC, Samsung Medical Centre; W/D, well differentiated; M/D, moderately differentiated; P/D, poorly differentiated; NA, not available

Table S2. Prediction of activated upstream regulators in the COD subtype.

Upstream Regulator	Exp Log Ratio	Molecule Type	Predicted Activation State	Activation z-score	P-value*
TGFB1		growth factor	Activated	3.912	6.35E-06
KDM5B		transcription regulator	Activated	2.934	2.59E-03
CBX5		transcription regulator	Activated	2.837	3.85E-05
TLR2		transmembrane receptor	Activated	2.599	3.59E-01
P38 MAPK		group	Activated	2.391	5.56E-02
GLI1	1.503	transcription regulator	Activated	2.365	3.82E-06
SNAI1		transcription regulator	Activated	2.354	2.37E-02
IgG		complex	Activated	2.289	1.91E-02
TWIST1		transcription regulator	Activated	2.243	3.48E-05
HR		transcription regulator	Activated	2.236	5.66E-02
E2F6		transcription regulator	Activated	2.236	4.84E-02
DEF6		other	Activated	2.229	7.82E-04
PELP1		other	Activated	2.219	1.74E-01
ETV5		transcription regulator	Activated	2.213	2.95E-06
HDAC3		transcription regulator	Activated	2.208	1.36E-03
MYOCD	1.769	transcription regulator	Activated	2.205	1.36E-06
HDAC6		transcription regulator	Activated	2.158	7.91E-04
POU2F2		transcription regulator	Activated	2.121	4.75E-02
TP53		transcription regulator	Activated	2.077	1.41E-01
CTR9		other	Activated	2	8.49E-02
CCL2	1.28	cytokine	Activated	2	8.49E-02
IGF1	2.35	growth factor	Activated	2	2.93E-02
SOX11		transcription regulator	Activated	2	2.45E-01
HELLS		enzyme	Inhibited	-2	8.79E-03
NCOA3		transcription regulator	Inhibited	-2.01	8.70E-05
CDH1	-1.015	other	Inhibited	-2.2	1.74E-01
mir-218		microrna	Inhibited	-2.394	9.68E-04
E2F1		transcription regulator	Inhibited	-2.408	2.33E-02
EHF	-1.239	transcription regulator	Inhibited	-2.688	1.55E-03
AREG	-1.085	growth factor	Inhibited	-2.709	1.66E-02
ESRRA		ligand-dependent nuclear receptor	Inhibited	-2.728	1.18E-03
ELAVL1		other	Inhibited	-2.813	3.19E-02
TP63		transcription regulator	Inhibited	-2.818	3.30E-03
WISP2	2.748	growth factor	Inhibited	-2.891	3.48E-05
FOXO1		transcription regulator	Inhibited	-2.941	1.28E-04
E2F3		transcription regulator	Inhibited	-3.051	1.17E-02
RABL6		other	Inhibited	-3.638	2.18E-05

*P-values were calculated by Fisher's exact test based on the number of interconnected genes with upstream regulators.

It was predicted that an upstream regulator was activated when the activated Z-score was greater than 0, while it was predicted that an upstream regulator was inhibited when the activated Z-score was less than 0. This prediction was carried out using Ingenuity Pathway Analysis software.

Abbreviations: COD, core diffuse type.

Table S3. Differentially mutated genes between INT and COD subtypes in diffuse type gastric cancer of the TCGA cohort (n = 61).

Gene symbol	Total mutation frequency (%)	Frequency of mutations in the INT subtype (n=26)	Frequency of mutations in the COD subtype (n=35)	P-value*
MUC16	31.15	14	5	1.74E-03
CSMD1	27.87	11	6	4.37E-02
FAT4	27.87	11	6	4.37E-02
ARID1A	21.31	11	2	9.78E-04
DMD	18.03	8	3	4.18E-02
GLI3	18.03	8	3	4.18E-02
MUC4	18.03	8	3	4.18E-02
PIK3CA	18.03	8	3	4.18E-02
PREX2	18.03	8	3	4.18E-02
SACS	18.03	8	3	4.18E-02
COL11A1	16.39	9	1	1.27E-03
XIST	16.39	9	1	1.27E-03
DST	16.39	8	2	1.36E-02
MUC6	16.39	8	2	1.36E-02
RYR2	16.39	8	2	1.36E-02
ZFHX4	16.39	8	2	1.36E-02
KMT2D	14.75	9	0	1.80E-04
VPS13B	14.75	9	0	1.80E-04
CSMD3	14.75	7	2	3.00E-02
CUBN	14.75	7	2	3.00E-02
FAT3	14.75	7	2	3.00E-02
HMCN1	14.75	7	2	3.00E-02
VCAN	14.75	7	2	3.00E-02
XIRP2	14.75	7	2	3.00E-02
ACVR2A	13.11	8	0	5.31E-04
ASPM	13.11	8	0	5.31E-04
ATP2B3	13.11	8	0	5.31E-04
HYDIN	13.11	8	0	5.31E-04
MDN1	13.11	8	0	5.31E-04
RNF213	13.11	8	0	5.31E-04
ATG2B	13.11	7	1	8.35E-03
CACNA1H	13.11	7	1	8.35E-03
DNAH8	13.11	7	1	8.35E-03
EP400	13.11	7	1	8.35E-03
GNAS	13.11	7	1	8.35E-03
LAMA1	13.11	7	1	8.35E-03
MEGF8	13.11	7	1	8.35E-03
NBEA	13.11	7	1	8.35E-03
RYR3	13.11	7	1	8.35E-03
ANK3	11.48	7	0	1.51E-03
CAMTA2	11.48	7	0	1.51E-03
CPSF1	11.48	7	0	1.51E-03
FLG	11.48	7	0	1.51E-03
KCNA4	11.48	7	0	1.51E-03
KIAA1161	11.48	7	0	1.51E-03
PXDNL	11.48	7	0	1.51E-03
SALL4	11.48	7	0	1.51E-03
TG	11.48	7	0	1.51E-03
WNK1	11.48	7	0	1.51E-03

ABCC8	11.48	6	1	3.54E-02
AHNAK	11.48	6	1	3.54E-02
CDH23	11.48	6	1	3.54E-02
CELSR3	11.48	6	1	3.54E-02
CNTN6	11.48	6	1	3.54E-02
DOCK3	11.48	6	1	3.54E-02
FAT1	11.48	6	1	3.54E-02
FREM2	11.48	6	1	3.54E-02
HECTD4	11.48	6	1	3.54E-02
IRS4	11.48	6	1	3.54E-02
LRP1	11.48	6	1	3.54E-02
MGAM	11.48	6	1	3.54E-02
NIPBL	11.48	6	1	3.54E-02
PCDH10	11.48	6	1	3.54E-02
PCDHA13	11.48	6	1	3.54E-02
PCDHB4	11.48	6	1	3.54E-02
PCDHGA2	11.48	6	1	3.54E-02
PKHD1	11.48	6	1	3.54E-02
PLEC	11.48	6	1	3.54E-02
SALL1	11.48	6	1	3.54E-02
ZBTB20	11.48	6	1	3.54E-02
ZEB1	11.48	6	1	3.54E-02
BDP1	9.84	6	0	4.15E-03
CENPF	9.84	6	0	4.15E-03
CEP164	9.84	6	0	4.15E-03
COL6A6	9.84	6	0	4.15E-03
CR2	9.84	6	0	4.15E-03
CREBBP	9.84	6	0	4.15E-03
FASN	9.84	6	0	4.15E-03
FBXL7	9.84	6	0	4.15E-03
HECW1	9.84	6	0	4.15E-03
HERC2	9.84	6	0	4.15E-03
ITGA7	9.84	6	0	4.15E-03
JARID2	9.84	6	0	4.15E-03
MALAT1	9.84	6	0	4.15E-03
MBD6	9.84	6	0	4.15E-03
MSH3	9.84	6	0	4.15E-03
MYBPC3	9.84	6	0	4.15E-03
NFASC	9.84	6	0	4.15E-03
NRXN2	9.84	6	0	4.15E-03
NUP214	9.84	6	0	4.15E-03
PLXNB1	9.84	6	0	4.15E-03
POTEC	9.84	6	0	4.15E-03
PTPRU	9.84	6	0	4.15E-03
RET	9.84	6	0	4.15E-03
SBNO1	9.84	6	0	4.15E-03
SEC31B	9.84	6	0	4.15E-03
SI	9.84	6	0	4.15E-03
SP100	9.84	6	0	4.15E-03
TCHH	9.84	6	0	4.15E-03
TRIO	9.84	6	0	4.15E-03
TSC2	9.84	6	0	4.15E-03
TUBGCP6	9.84	6	0	4.15E-03

XYLT2	9.84	6	0	4.15E-03
ZC3H18	9.84	6	0	4.15E-03
ABCA7	8.2	5	0	1.11E-02
ABCB5	8.2	5	0	1.11E-02
ADAMTS2	8.2	5	0	1.11E-02
ADCY8	8.2	5	0	1.11E-02
ADD2	8.2	5	0	1.11E-02
AKAP11	8.2	5	0	1.11E-02
AKAP9	8.2	5	0	1.11E-02
AMPD3	8.2	5	0	1.11E-02
ANKRD35	8.2	5	0	1.11E-02
ARHGAP5	8.2	5	0	1.11E-02
ARHGEF11	8.2	5	0	1.11E-02
ASH1L	8.2	5	0	1.11E-02
ATP2A3	8.2	5	0	1.11E-02
BAX	8.2	5	0	1.11E-02
BRCA2	8.2	5	0	1.11E-02
CEP250	8.2	5	0	1.11E-02
CHD2	8.2	5	0	1.11E-02
CNTN5	8.2	5	0	1.11E-02
CPAMD8	8.2	5	0	1.11E-02
CUL9	8.2	5	0	1.11E-02
CUX1	8.2	5	0	1.11E-02
DAPK1	8.2	5	0	1.11E-02
DCAF13	8.2	5	0	1.11E-02
DIDO1	8.2	5	0	1.11E-02
DMXL1	8.2	5	0	1.11E-02
DOCK7	8.2	5	0	1.11E-02
ELMSAN1	8.2	5	0	1.11E-02
EPG5	8.2	5	0	1.11E-02
EPHA2	8.2	5	0	1.11E-02
EPHA5	8.2	5	0	1.11E-02
FBN2	8.2	5	0	1.11E-02
GPRASP1	8.2	5	0	1.11E-02
GRIN2B	8.2	5	0	1.11E-02
GRM2	8.2	5	0	1.11E-02
GZF1	8.2	5	0	1.11E-02
HSPA12A	8.2	5	0	1.11E-02
HUWE1	8.2	5	0	1.11E-02
INF2	8.2	5	0	1.11E-02
INTS1	8.2	5	0	1.11E-02
ITGA2B	8.2	5	0	1.11E-02
ITPR3	8.2	5	0	1.11E-02
KCND2	8.2	5	0	1.11E-02
KDM5B	8.2	5	0	1.11E-02
KNDC1	8.2	5	0	1.11E-02
KNOP1	8.2	5	0	1.11E-02
LAMA4	8.2	5	0	1.11E-02
LARP4B	8.2	5	0	1.11E-02
MLLT4	8.2	5	0	1.11E-02
MSH6	8.2	5	0	1.11E-02
MTUS2	8.2	5	0	1.11E-02
MXRA8	8.2	5	0	1.11E-02

MYBL1	8.2	5	0	1.11E-02
MYCBP2	8.2	5	0	1.11E-02
MYH3	8.2	5	0	1.11E-02
MYH6	8.2	5	0	1.11E-02
MYO16	8.2	5	0	1.11E-02
NOTCH4	8.2	5	0	1.11E-02
NPAP1	8.2	5	0	1.11E-02
NUP188	8.2	5	0	1.11E-02
OBSL1	8.2	5	0	1.11E-02
OR5M1	8.2	5	0	1.11E-02
PBRM1	8.2	5	0	1.11E-02
PCNT	8.2	5	0	1.11E-02
PDZD2	8.2	5	0	1.11E-02
PEG3	8.2	5	0	1.11E-02
PKD1L1	8.2	5	0	1.11E-02
PML	8.2	5	0	1.11E-02
PPFIA3	8.2	5	0	1.11E-02
PRDM16	8.2	5	0	1.11E-02
RERE	8.2	5	0	1.11E-02
ROCK1	8.2	5	0	1.11E-02
SAMD9L	8.2	5	0	1.11E-02
SAP130	8.2	5	0	1.11E-02
SDHAP1	8.2	5	0	1.11E-02
SEMA5B	8.2	5	0	1.11E-02
SP140	8.2	5	0	1.11E-02
STAT2	8.2	5	0	1.11E-02
SUSD2	8.2	5	0	1.11E-02
TIE1	8.2	5	0	1.11E-02
TMEM132D	8.2	5	0	1.11E-02
TNK2	8.2	5	0	1.11E-02
TP53BP1	8.2	5	0	1.11E-02
TRANK1	8.2	5	0	1.11E-02
TRPM2	8.2	5	0	1.11E-02
TVP23C	8.2	5	0	1.11E-02
UBE2O	8.2	5	0	1.11E-02
USP48	8.2	5	0	1.11E-02
WFIKKN2	8.2	5	0	1.11E-02
ZFR	8.2	5	0	1.11E-02
ZNF236	8.2	5	0	1.11E-02
ZNF835	8.2	5	0	1.11E-02
ABCA1	6.56	4	0	2.86E-02
ADAM23	6.56	4	0	2.86E-02
ADAMTS7	6.56	4	0	2.86E-02
ADAMTSL1	6.56	4	0	2.86E-02
ADAMTSL3	6.56	4	0	2.86E-02
ADCY9	6.56	4	0	2.86E-02
AEBP1	6.56	4	0	2.86E-02
AGO2	6.56	4	0	2.86E-02
AGRN	6.56	4	0	2.86E-02
ALDH1A3	6.56	4	0	2.86E-02
ALOX5	6.56	4	0	2.86E-02
AMZ1	6.56	4	0	2.86E-02
APC2	6.56	4	0	2.86E-02

ARFGEF1	6.56	4	0	2.86E-02
ARID2	6.56	4	0	2.86E-02
ATP4A	6.56	4	0	2.86E-02
ATRNL1	6.56	4	0	2.86E-02
AXIN1	6.56	4	0	2.86E-02
AXL	6.56	4	0	2.86E-02
B4GALT7	6.56	4	0	2.86E-02
BAZ2B	6.56	4	0	2.86E-02
BCORL1	6.56	4	0	2.86E-02
BLM	6.56	4	0	2.86E-02
BTBD11	6.56	4	0	2.86E-02
C2CD3	6.56	4	0	2.86E-02
CABIN1	6.56	4	0	2.86E-02
CACNA1B	6.56	4	0	2.86E-02
CACNA1G	6.56	4	0	2.86E-02
CACNA2D1	6.56	4	0	2.86E-02
CASD1	6.56	4	0	2.86E-02
CASR	6.56	4	0	2.86E-02
CASZ1	6.56	4	0	2.86E-02
CCDC178	6.56	4	0	2.86E-02
CCDC38	6.56	4	0	2.86E-02
CCKBR	6.56	4	0	2.86E-02
CD4	6.56	4	0	2.86E-02
CDAN1	6.56	4	0	2.86E-02
CDH10	6.56	4	0	2.86E-02
CENPE	6.56	4	0	2.86E-02
CHD4	6.56	4	0	2.86E-02
CHD5	6.56	4	0	2.86E-02
CLUH	6.56	4	0	2.86E-02
COL11A2	6.56	4	0	2.86E-02
COL24A1	6.56	4	0	2.86E-02
COL4A1	6.56	4	0	2.86E-02
COL5A2	6.56	4	0	2.86E-02
COL9A3	6.56	4	0	2.86E-02
CPD	6.56	4	0	2.86E-02
CPEB2	6.56	4	0	2.86E-02
CRAT	6.56	4	0	2.86E-02
CRYGA	6.56	4	0	2.86E-02
CTSW	6.56	4	0	2.86E-02
CYP19A1	6.56	4	0	2.86E-02
DCAF12L2	6.56	4	0	2.86E-02
DCAF4L2	6.56	4	0	2.86E-02
DCC	6.56	4	0	2.86E-02
DDX59	6.56	4	0	2.86E-02
DENND2A	6.56	4	0	2.86E-02
DENND5A	6.56	4	0	2.86E-02
DGKB	6.56	4	0	2.86E-02
DHX35	6.56	4	0	2.86E-02
DLEC1	6.56	4	0	2.86E-02
DMBT1	6.56	4	0	2.86E-02
DNAH17	6.56	4	0	2.86E-02
DNM2	6.56	4	0	2.86E-02
DPEP1	6.56	4	0	2.86E-02

DPYSL4	6.56	4	0	2.86E-02
DROSHA	6.56	4	0	2.86E-02
DSCAM	6.56	4	0	2.86E-02
DYRK1B	6.56	4	0	2.86E-02
EFCAB5	6.56	4	0	2.86E-02
EIF2B4	6.56	4	0	2.86E-02
EIF4G3	6.56	4	0	2.86E-02
EIF5B	6.56	4	0	2.86E-02
ELAVL4	6.56	4	0	2.86E-02
EPHB1	6.56	4	0	2.86E-02
EPRS	6.56	4	0	2.86E-02
ERBB3	6.56	4	0	2.86E-02
ERCC6	6.56	4	0	2.86E-02
EVPL	6.56	4	0	2.86E-02
FAM135A	6.56	4	0	2.86E-02
FAM171B	6.56	4	0	2.86E-02
FAM184A	6.56	4	0	2.86E-02
FAM193A	6.56	4	0	2.86E-02
FAM208B	6.56	4	0	2.86E-02
FAM214B	6.56	4	0	2.86E-02
FARP1	6.56	4	0	2.86E-02
FBXL18	6.56	4	0	2.86E-02
FER1L6	6.56	4	0	2.86E-02
FGD1	6.56	4	0	2.86E-02
FGFR1	6.56	4	0	2.86E-02
FGFR2	6.56	4	0	2.86E-02
FIGN	6.56	4	0	2.86E-02
FKBP10	6.56	4	0	2.86E-02
FLNB	6.56	4	0	2.86E-02
FLT4	6.56	4	0	2.86E-02
GDF10	6.56	4	0	2.86E-02
GLI1	6.56	4	0	2.86E-02
GLI2	6.56	4	0	2.86E-02
GPR158	6.56	4	0	2.86E-02
GPR45	6.56	4	0	2.86E-02
GRID1	6.56	4	0	2.86E-02
HDAC4	6.56	4	0	2.86E-02
HEATR1	6.56	4	0	2.86E-02
HIVEP2	6.56	4	0	2.86E-02
HNF1A	6.56	4	0	2.86E-02
HRC	6.56	4	0	2.86E-02
IFT140	6.56	4	0	2.86E-02
INVS	6.56	4	0	2.86E-02
IRX6	6.56	4	0	2.86E-02
ITGA3	6.56	4	0	2.86E-02
ITGA6	6.56	4	0	2.86E-02
ITGB1	6.56	4	0	2.86E-02
JAG2	6.56	4	0	2.86E-02
KCNC2	6.56	4	0	2.86E-02
KCNH2	6.56	4	0	2.86E-02
KDM2B	6.56	4	0	2.86E-02
KIAA1407	6.56	4	0	2.86E-02
KIAA1683	6.56	4	0	2.86E-02

KIDINS220	6.56	4	0	2.86E-02
KIF1B	6.56	4	0	2.86E-02
KIF26B	6.56	4	0	2.86E-02
KLHL40	6.56	4	0	2.86E-02
KMT2A	6.56	4	0	2.86E-02
KNTC1	6.56	4	0	2.86E-02
KREMEN1	6.56	4	0	2.86E-02
LAMB2	6.56	4	0	2.86E-02
LAMC1	6.56	4	0	2.86E-02
LIG3	6.56	4	0	2.86E-02
LOC100134368	6.56	4	0	2.86E-02
LPPR4	6.56	4	0	2.86E-02
LRFN3	6.56	4	0	2.86E-02
LRP5	6.56	4	0	2.86E-02
LRRC52	6.56	4	0	2.86E-02
LTBP3	6.56	4	0	2.86E-02
MAP3K4	6.56	4	0	2.86E-02
MAST2	6.56	4	0	2.86E-02
MDC1	6.56	4	0	2.86E-02
MDGA1	6.56	4	0	2.86E-02
MDH1B	6.56	4	0	2.86E-02
MECOM	6.56	4	0	2.86E-02
MICAL3	6.56	4	0	2.86E-02
MIOS	6.56	4	0	2.86E-02
MKL1	6.56	4	0	2.86E-02
MMRN2	6.56	4	0	2.86E-02
MTF2	6.56	4	0	2.86E-02
MVK	6.56	4	0	2.86E-02
MYH14	6.56	4	0	2.86E-02
MYH9	6.56	4	0	2.86E-02
MYO1A	6.56	4	0	2.86E-02
MYO7A	6.56	4	0	2.86E-02
NCOR2	6.56	4	0	2.86E-02
NEB	6.56	4	0	2.86E-02
NEU2	6.56	4	0	2.86E-02
NEUROD2	6.56	4	0	2.86E-02
NFATC2	6.56	4	0	2.86E-02
NLRC5	6.56	4	0	2.86E-02
NLRP8	6.56	4	0	2.86E-02
NPHP4	6.56	4	0	2.86E-02
NR4A2	6.56	4	0	2.86E-02
NRAP	6.56	4	0	2.86E-02
NRXN3	6.56	4	0	2.86E-02
NSD1	6.56	4	0	2.86E-02
NUP210	6.56	4	0	2.86E-02
OSBP2	6.56	4	0	2.86E-02
OTOA	6.56	4	0	2.86E-02
OTOF	6.56	4	0	2.86E-02
P2RY6	6.56	4	0	2.86E-02
PAX1	6.56	4	0	2.86E-02
PCDHA1	6.56	4	0	2.86E-02
PCDHA7	6.56	4	0	2.86E-02
PCDHA8	6.56	4	0	2.86E-02

PCDHA9	6.56	4	0	2.86E-02
PCDHGC3	6.56	4	0	2.86E-02
PDCD11	6.56	4	0	2.86E-02
PDS5B	6.56	4	0	2.86E-02
PITPNM1	6.56	4	0	2.86E-02
PNPLA7	6.56	4	0	2.86E-02
POSTN	6.56	4	0	2.86E-02
PRDM15	6.56	4	0	2.86E-02
PRDX3	6.56	4	0	2.86E-02
PRF1	6.56	4	0	2.86E-02
PROS1	6.56	4	0	2.86E-02
PRR12	6.56	4	0	2.86E-02
PRRC2C	6.56	4	0	2.86E-02
PSD	6.56	4	0	2.86E-02
PSD2	6.56	4	0	2.86E-02
PSME4	6.56	4	0	2.86E-02
PTP4A2	6.56	4	0	2.86E-02
PTPRF	6.56	4	0	2.86E-02
PTPRJ	6.56	4	0	2.86E-02
PTPRR	6.56	4	0	2.86E-02
PTPRS	6.56	4	0	2.86E-02
RAI1	6.56	4	0	2.86E-02
RANBP2	6.56	4	0	2.86E-02
RBM24	6.56	4	0	2.86E-02
RGS12	6.56	4	0	2.86E-02
RHOBTB2	6.56	4	0	2.86E-02
RLTPR	6.56	4	0	2.86E-02
RNF216	6.56	4	0	2.86E-02
ROBO3	6.56	4	0	2.86E-02
ROR2	6.56	4	0	2.86E-02
RREB1	6.56	4	0	2.86E-02
SCLT1	6.56	4	0	2.86E-02
SCN5A	6.56	4	0	2.86E-02
SDCBP2	6.56	4	0	2.86E-02
SDK2	6.56	4	0	2.86E-02
SEC16A	6.56	4	0	2.86E-02
SEC23IP	6.56	4	0	2.86E-02
SETDB1	6.56	4	0	2.86E-02
SGOL1	6.56	4	0	2.86E-02
SGSM1	6.56	4	0	2.86E-02
SHANK1	6.56	4	0	2.86E-02
SIGLEC5	6.56	4	0	2.86E-02
SLC24A3	6.56	4	0	2.86E-02
SLC2A7	6.56	4	0	2.86E-02
SLC35F5	6.56	4	0	2.86E-02
SLC46A3	6.56	4	0	2.86E-02
SLC6A3	6.56	4	0	2.86E-02
SLIT3	6.56	4	0	2.86E-02
SMG5	6.56	4	0	2.86E-02
SMG6	6.56	4	0	2.86E-02
SMTN	6.56	4	0	2.86E-02
SND1	6.56	4	0	2.86E-02
SORCS2	6.56	4	0	2.86E-02

SORL1	6.56	4	0	2.86E-02
SPG20	6.56	4	0	2.86E-02
SPHKAP	6.56	4	0	2.86E-02
SPRED2	6.56	4	0	2.86E-02
SRPR	6.56	4	0	2.86E-02
ST18	6.56	4	0	2.86E-02
SUPT6H	6.56	4	0	2.86E-02
SYBU	6.56	4	0	2.86E-02
SYTL2	6.56	4	0	2.86E-02
TAF1	6.56	4	0	2.86E-02
TBC1D2	6.56	4	0	2.86E-02
TCERG1	6.56	4	0	2.86E-02
TDRD6	6.56	4	0	2.86E-02
TENM2	6.56	4	0	2.86E-02
TESK1	6.56	4	0	2.86E-02
TET3	6.56	4	0	2.86E-02
TEX14	6.56	4	0	2.86E-02
TF	6.56	4	0	2.86E-02
TMCC2	6.56	4	0	2.86E-02
TNIK	6.56	4	0	2.86E-02
TNKS	6.56	4	0	2.86E-02
TNN	6.56	4	0	2.86E-02
TNS3	6.56	4	0	2.86E-02
TPTE	6.56	4	0	2.86E-02
TRHDE	6.56	4	0	2.86E-02
TRIM55	6.56	4	0	2.86E-02
TRRAP	6.56	4	0	2.86E-02
TULP4	6.56	4	0	2.86E-02
ULK1	6.56	4	0	2.86E-02
UPP2	6.56	4	0	2.86E-02
USP42	6.56	4	0	2.86E-02
USP5	6.56	4	0	2.86E-02
VCP	6.56	4	0	2.86E-02
WDR62	6.56	4	0	2.86E-02
WDR72	6.56	4	0	2.86E-02
XDH	6.56	4	0	2.86E-02
XPNPEP1	6.56	4	0	2.86E-02
ZBTB7C	6.56	4	0	2.86E-02
ZC3H12A	6.56	4	0	2.86E-02
ZC3H3	6.56	4	0	2.86E-02
ZFHX3	6.56	4	0	2.86E-02
ZFPM2	6.56	4	0	2.86E-02
ZFYVE9	6.56	4	0	2.86E-02
ZMYM1	6.56	4	0	2.86E-02
ZNF227	6.56	4	0	2.86E-02
ZNF366	6.56	4	0	2.86E-02
ZNF407	6.56	4	0	2.86E-02
ZNF648	6.56	4	0	2.86E-02
ZNF667	6.56	4	0	2.86E-02
ZNRF3	6.56	4	0	2.86E-02

*P-values were obtained by Fisher's exact tests.
Abbreviations: INT, intestinal-like; COD, core diffuse type.

Supplementary figures

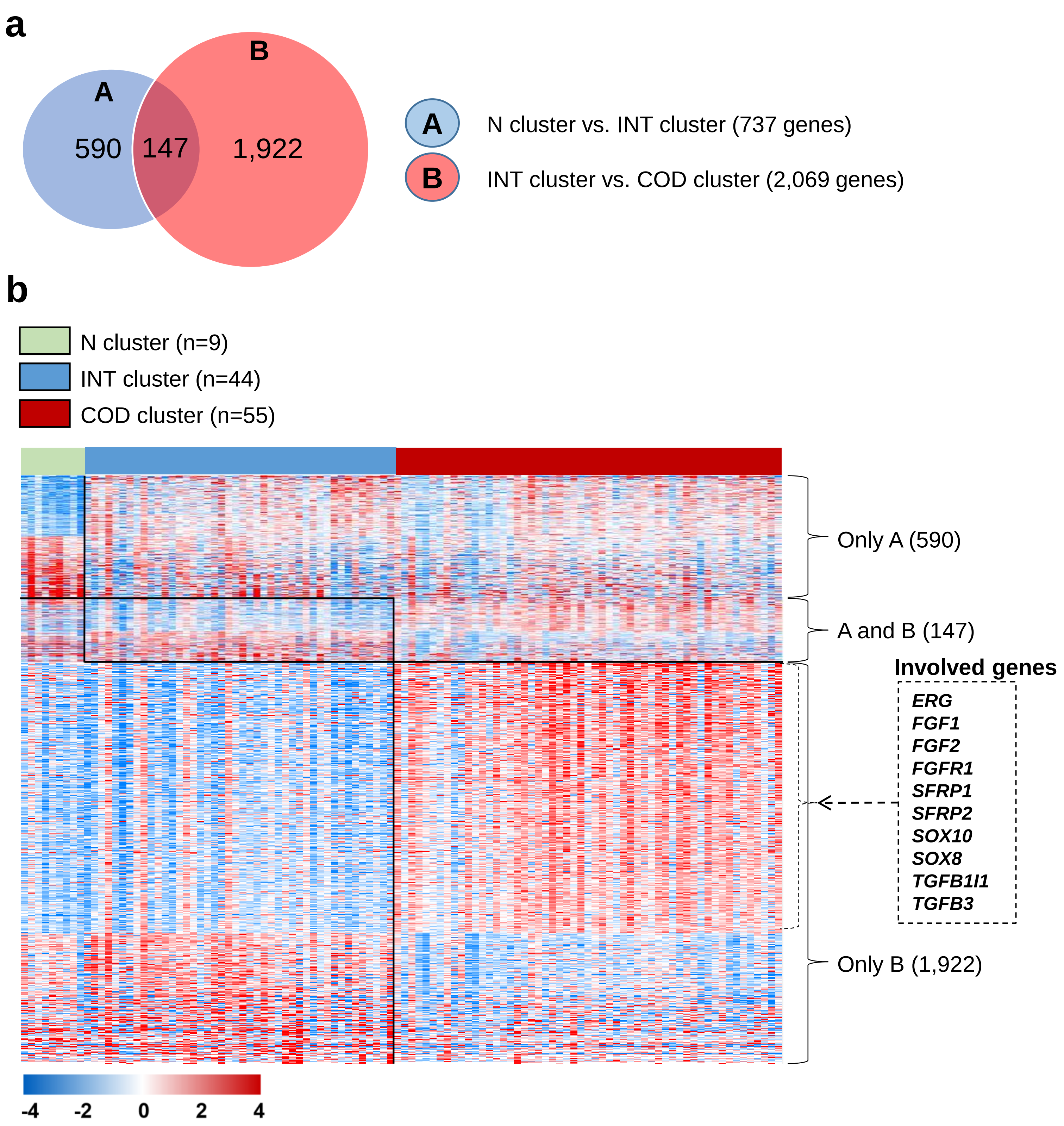

Figure S1. Comparative analysis of differentially expressed genes among the molecular subtypes of diffuse type gastric cancer (GC).

a Venn diagram of genes selected by the exact test using EdgeR software in the distinct sub patient groups. Genes in the blue circle (gene list A) represent those differentially expressed between normal-like (N) and intestinal-like (INT) subgroups. Genes in the red circle (gene list B) represent those differentially expressed between INT and core diffuse type (COD) subgroups. Cut-off criteria of a *P*-value of less than 0.001 and a 2-fold or greater relative difference were applied to select genes whose expression were significantly different between the two subtypes. **b** Expression patterns of selected genes in the Venn diagram. The data are presented in matrix format, in which rows represent individual genes and columns represent each tissue. The red and blue colors reflect high and low expression levels, respectively.

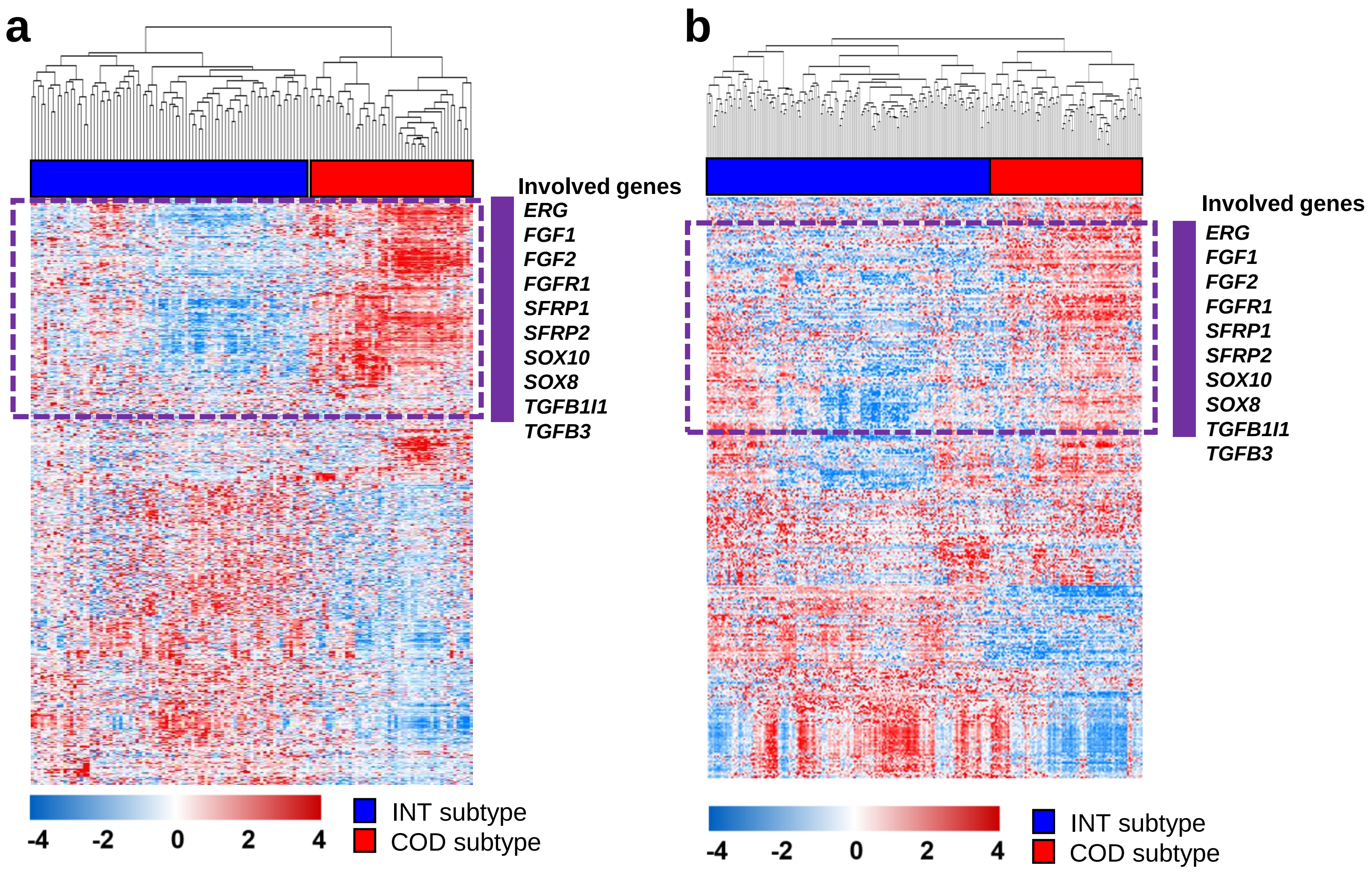

Figure S2. Gene expression pattern of the COD signature in patients with diffuse type gastric cancer in the ACRG (n=135) and SMC (n=267) cohorts.

a Gene expression pattern of the signature in the ACRG cohort. Through hierarchical clustering analysis, diffuse-type GC patients were divided into two groups: intestinal-like (INT) and diffuse-like (COD) subtypes. **b** Gene expression pattern of the signature in the SMC cohort. INT, intestinal-like; COD, core diffuse type.

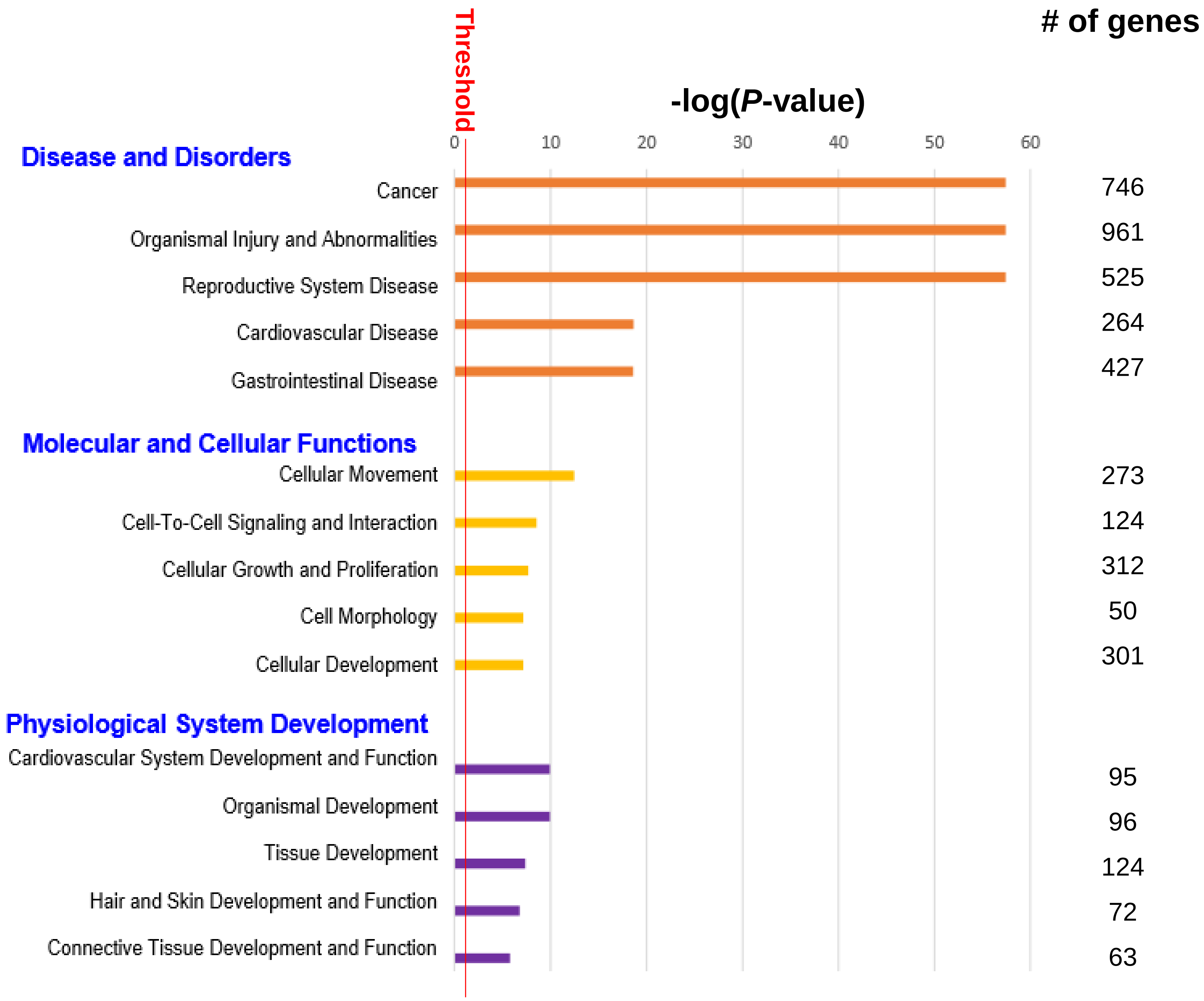

Figure S3. Gene set enrichments analysis of 1,922 genes associated with the COD-signature.

Classification enrichment was determined using Ingenuity Pathway Analysis software. The threshold of significance was $P < 0.05$. COD, core diffuse type.

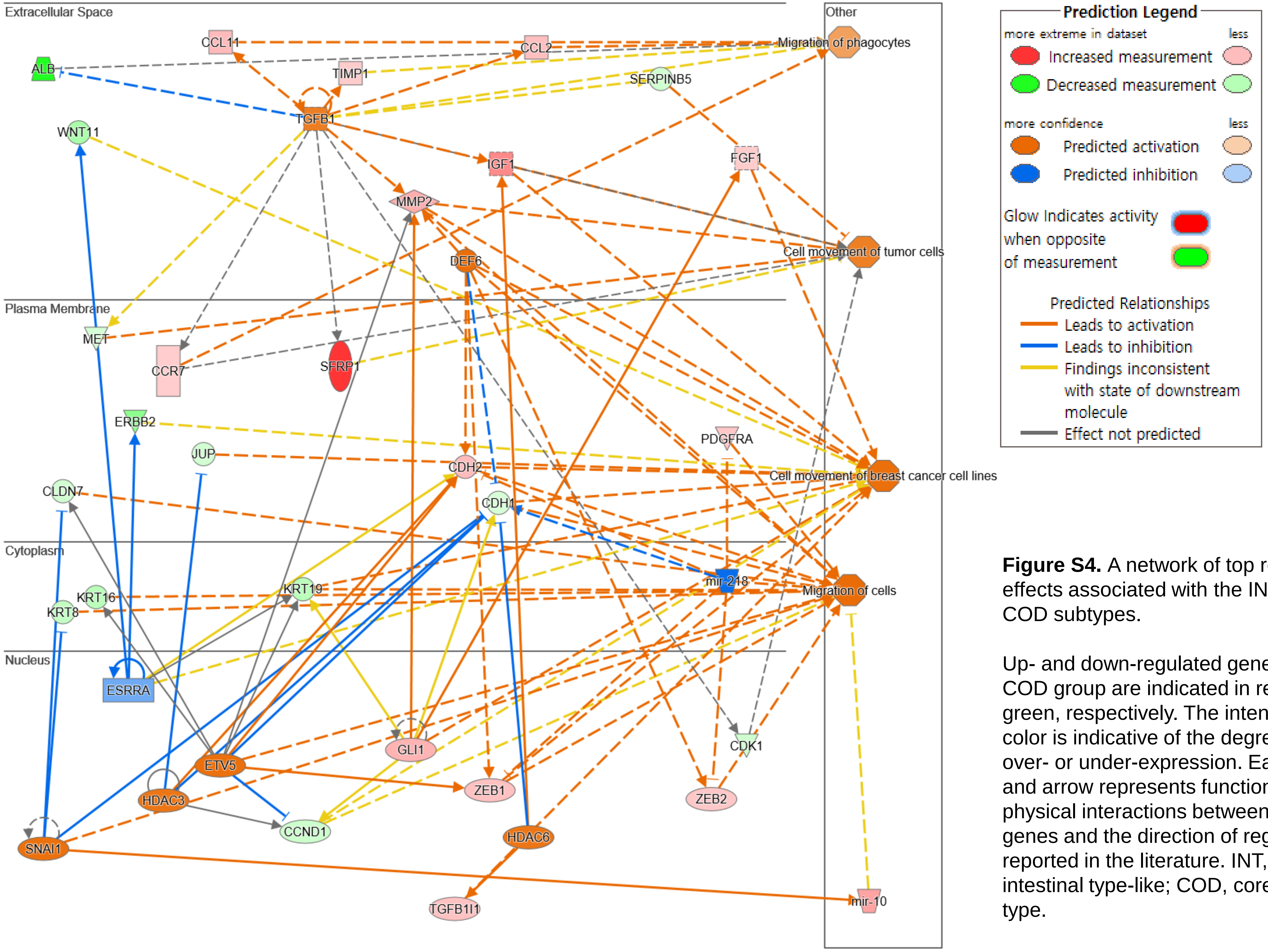

Figure S4. A network of top regular effects associated with the INT and COD subtypes.

Up- and down-regulated genes in the COD group are indicated in red and green, respectively. The intensity of color is indicative of the degree of over- or under-expression. Each line and arrow represents functional and physical interactions between the genes and the direction of regulation reported in the literature. INT, intestinal type-like; COD, core diffuse type.

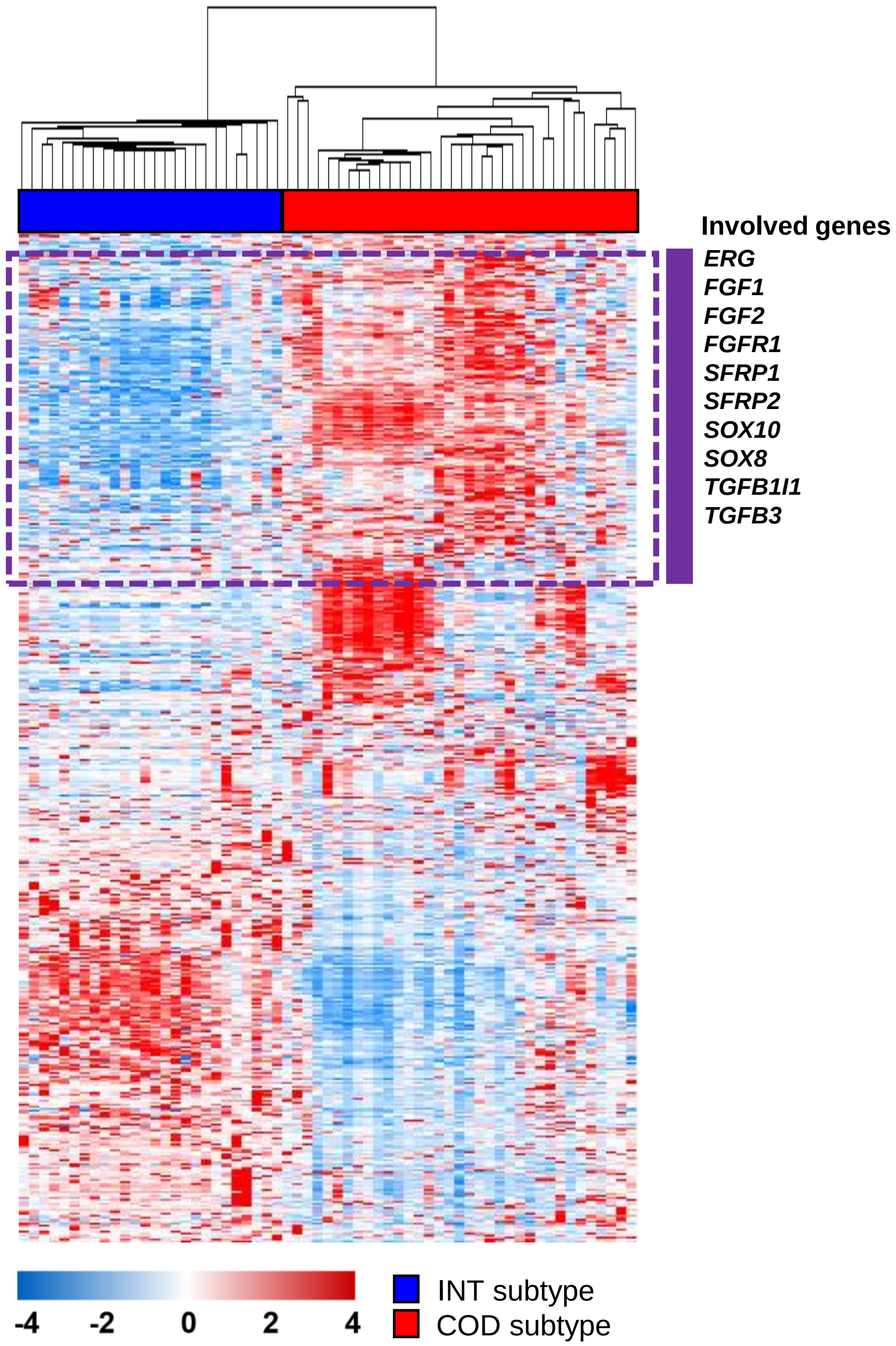

Figure S5. Gene expression pattern of the COD signature in patients with diffuse type gastric cancer of the TCGA cohort (n=61).

A total of the 1,716 genes associated with the COD signature were applied into a cluster analysis. INT, intestinal-like; COD, core diffuse type.

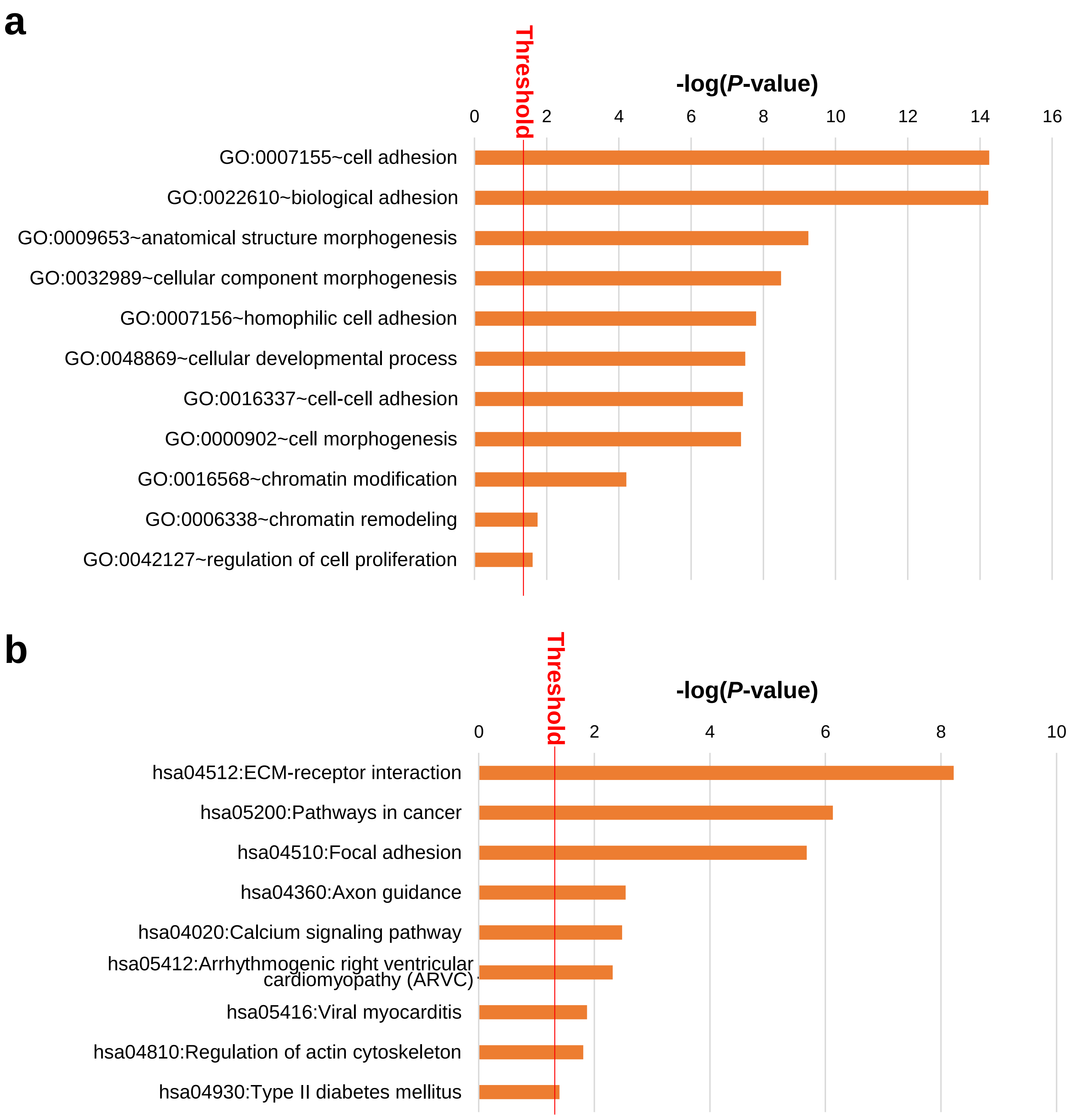

Figure S6. Function enrichment tests of differentially mutated genes between INT and COD subtypes in diffuse type gastric cancer of the TCGA cohort.

Significantly enriched functions were displayed in Gene Ontology Biological Process (**a**) or KEGG pathway (**b**) categories. *P*-values were obtained by Fisher's exact tests. INT, intestinal-like; COD, core diffuse type.

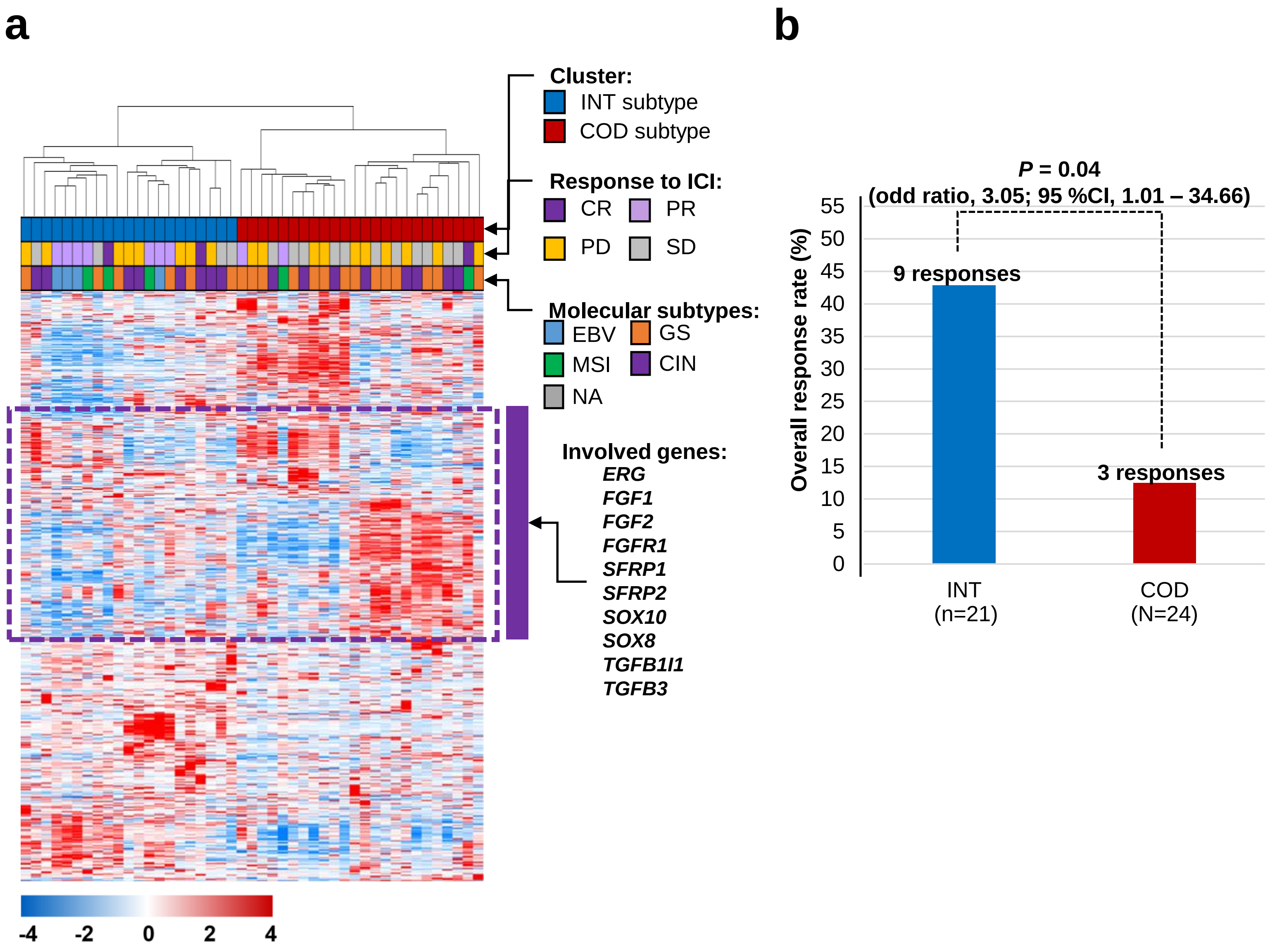

Figure S7. Predictive value of the COD signature for predicting response to immune checkpoint inhibitor (ICI) in gastric cancer (GC).

Gene expression data from a cohort of GC patients who were treated with an anti-PD-L1 agent (pembrolizumab) was used to estimate an association between COD signature and response to ICI (n=45). Gene expression data generated from RNA-seq are available at European Nucleotide Archive (ENA) under accession number PRJEB25780. **a** Gene expression pattern of the COD signature in GC. 1,803 genes associated with the COD signature were applied into a hierarchical clustering analysis, by which the patients were divided into two groups: intestinal-like (INT) and core diffuse-type (COD) subtypes. **b** Significance test comparing INT and COD subtypes in overall response rate (ORR) to ICI treatment. ORR of the INT subtype was significantly higher than that of the COD subtype (Fisher exact test; P = 0.04), indicating that patients in the INT subtype had a clinical benefit for ICI treatment than those in the COD subtype. CR, complete response; PR, partial response; PD, progressive disease; SD, stable disease; EBV, Epstein–Barr virus positive; MSI, microsatellite instable; GS, genome stable; CIN, chromosomal instability.

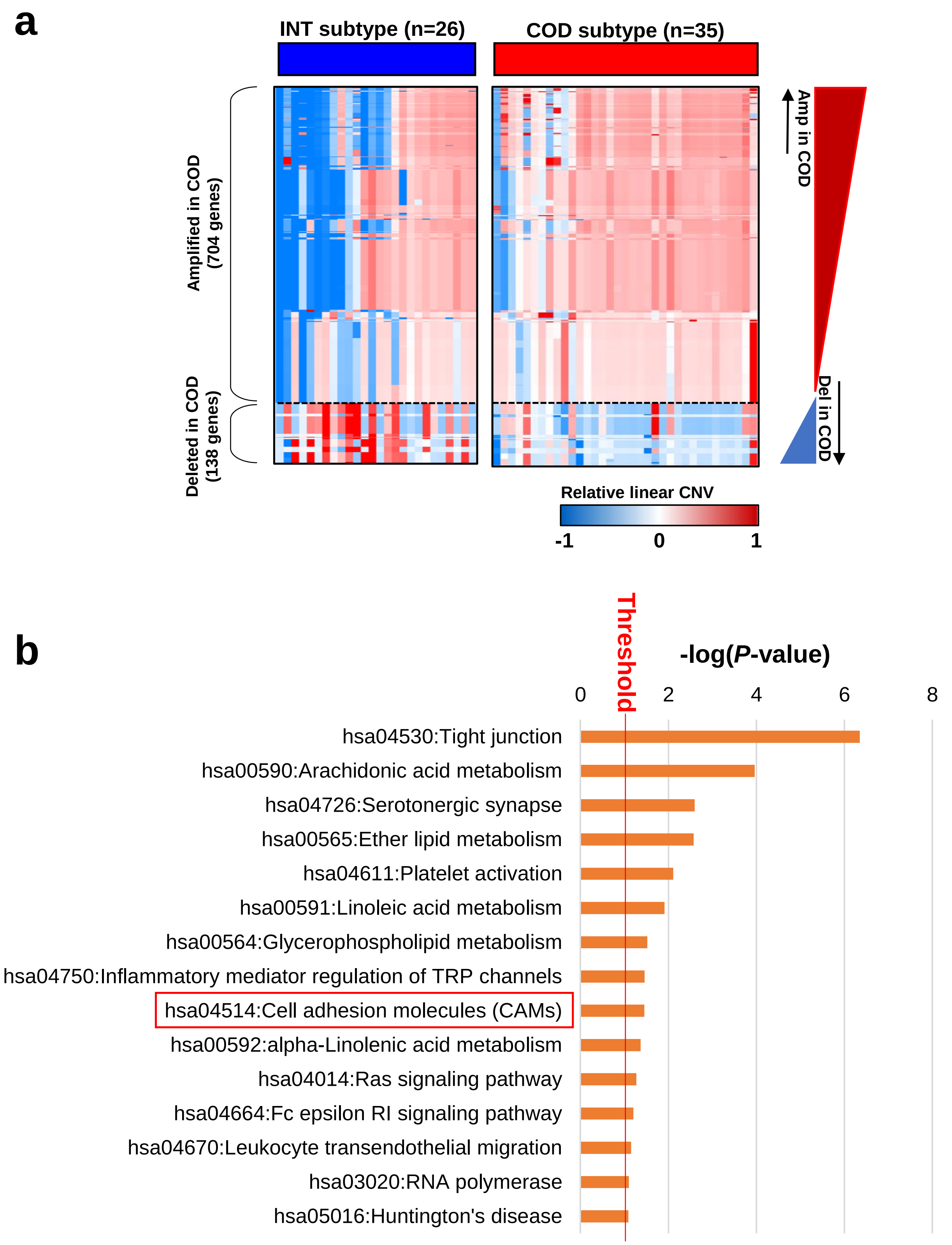

Figure S8. Copy number variation (CNV) pattern of the COD signature in patients with diffuse type gastric cancer (GC) in the TCGA cohort (n = 61).

a CNV patterns between diffuse-type GC patients with intestinal-like (INT) and core-diffuse (COD) subtypes. A total of 842 genes having statistically significant difference in CNV were selected (two-sample t-tests, $P < 0.01$). For estimating CNV difference, relative linear CNVs for each gene generated from the Affymetrix SNP6 platform were obtained. **b** Function enrichment test of genes with significant CNV difference between INT and COD subtypes in diffuse type GC. Significantly enriched functions were displayed in KEGG pathway. *P*-values were obtained by Fisher’s exact tests. INT, intestinal-like; COD, core diffuse type.

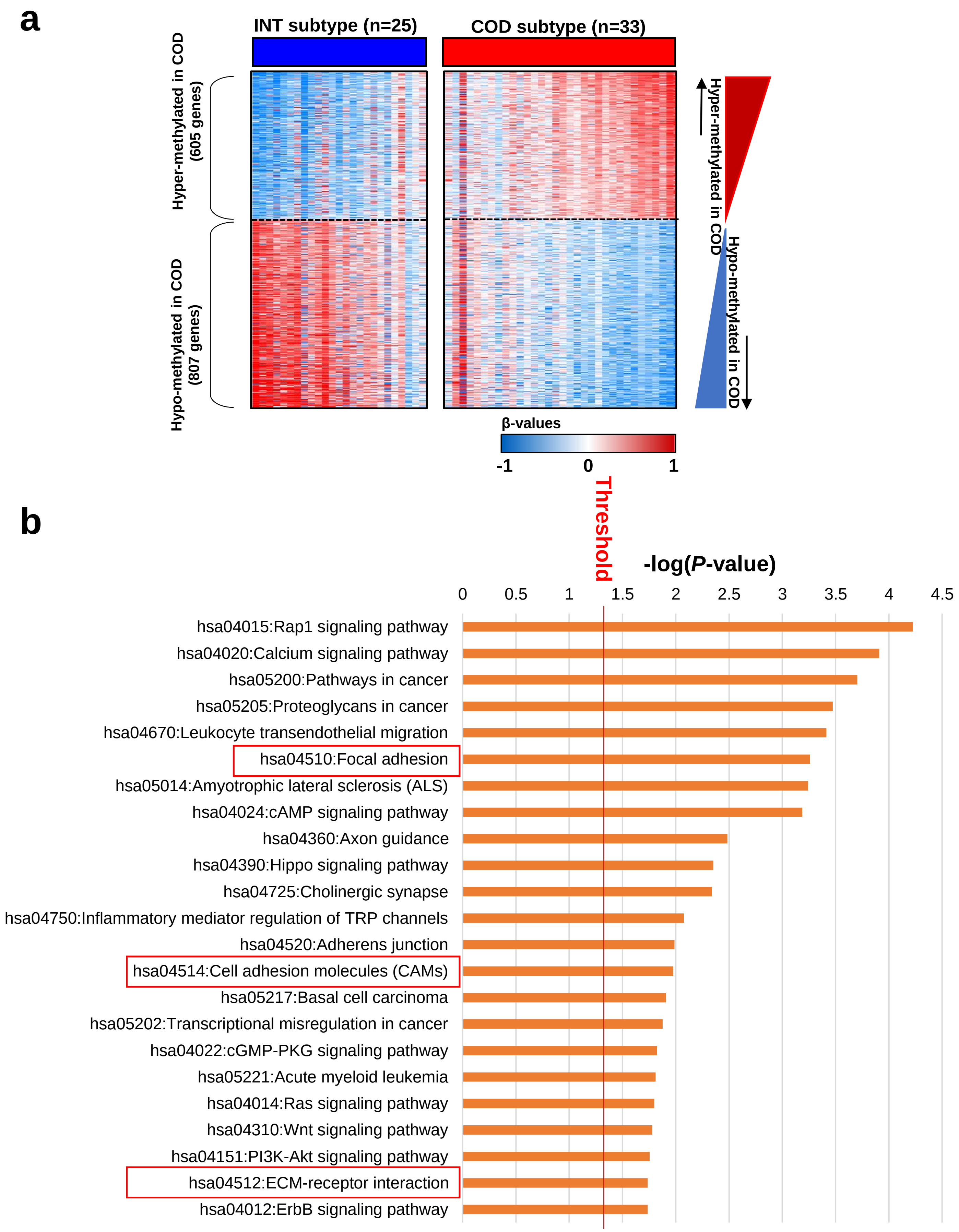

Figure S9. Methylation pattern of the COD signature in patients with diffuse type gastric cancer (GC) in the TCGA cohort (n = 58).

a Methylation patterns between diffuse-type GC patients with intestinal-like (INT) and core-diffuse (COD) subtypes. A total of 1,412 genes having statistically significant difference in methylation were selected (Wilcoxon rank sum tFor estimating methylation difference, β-values for each gene generated from the Illumina 450K array platform were obtained. ests, $P < 0.001$, $\triangle\beta > 0.15$). **b** Function enrichment test of genes with significant methylation difference between INT and COD subtypes in diffuse type GC. Significantly enriched functions were displayed in KEGG pathway. *P*-values were obtained by Fisher's exact tests. INT, intestinal-like; COD, core diffuse type.

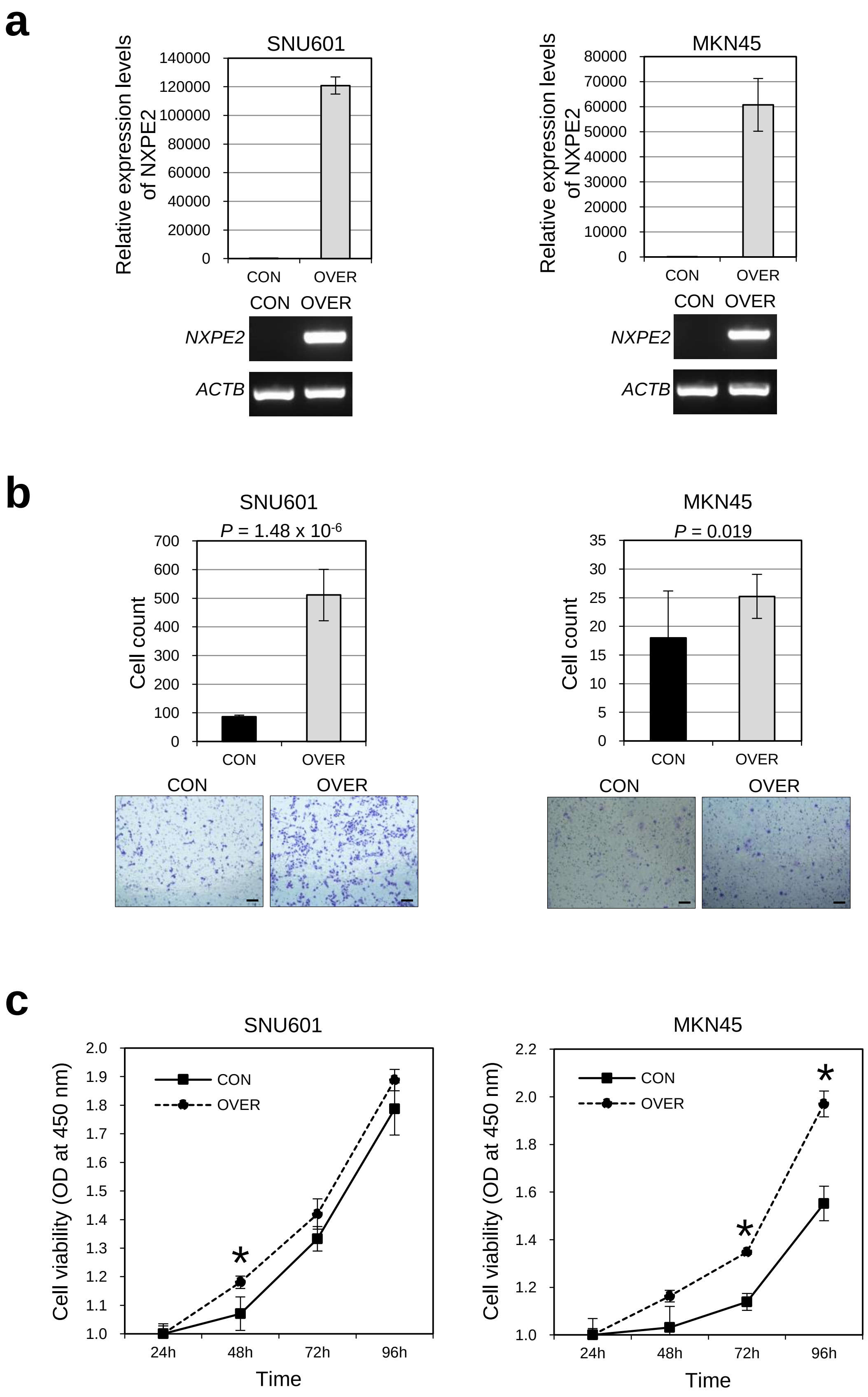

Figure S10. Promotion of DGC cell migration and cell proliferation by NXPE2 *in vitro*.

After ectopic NXPE2 overexpression: RT-PCR (**a**), migration (**b**), and proliferation (**c**) assays. Data are representative of three independent experiments. The error bars represent the standard error of the mean. *, $P < 0.05$.

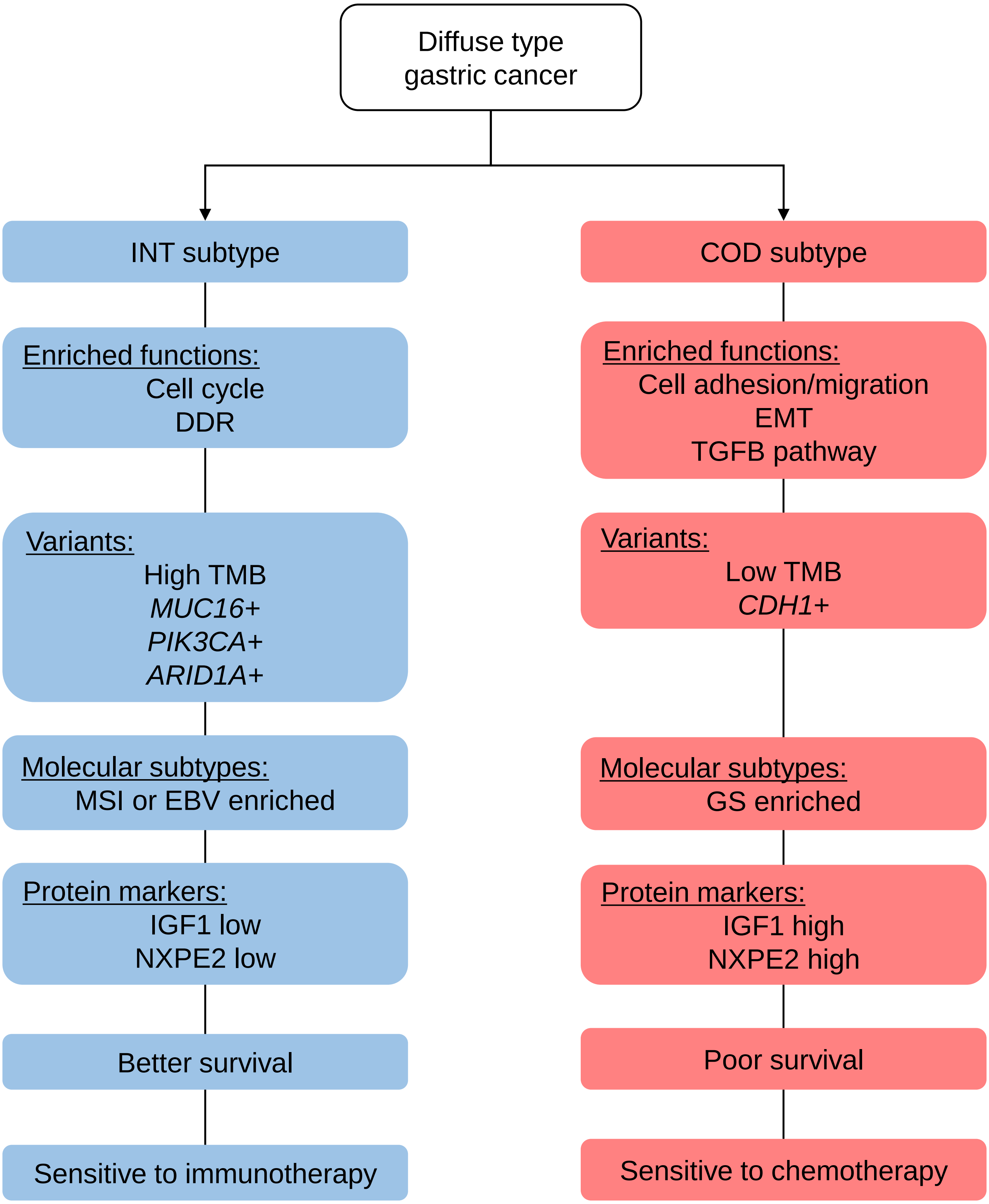

Figure S11. Schematic diagram of characteristics of INT and COD subtypes in diffuse type gastric cancer.

INT, intestinal-like; COD, core diffuse type; DDR, DNA damage response; EMT, epithelial-to-mesenchymal transition; TMB, tumour mutation burden; EBV, Epstein–Barr virus positive; MSI, microsatellite instable; GS, genome stable.
